# Polyethylenimine-based theranostic nanoplatform for glioma-targeting single-photon emission computed tomography imaging and anticancer drug delivery

**DOI:** 10.1186/s12951-020-00705-3

**Published:** 2020-10-14

**Authors:** Lingzhou Zhao, Jingyi Zhu, Jiali Gong, Ningning Song, Shan Wu, Wenli Qiao, Jiqin Yang, Meilin Zhu, Jinhua Zhao

**Affiliations:** 1 Department of Nuclear Medicine, Shanghai General Hospital, Shanghai Jiao Tong University School of Medicine, Shanghai, 200080 People’s Republic of China; 2 School of Pharmaceutical Sciences, Nanjing Tech University, Nanjing, 211816 People’s Republic of China; 3 Department of Nuclear Medicine, General Hospital of Ningxia Medical University, Yinchuan, 750004 Ningxia People’s Republic of China; 4 School of Basic Medical Sciences, Ningxia Medical University, Yinchuan, 750004 Ningxia People’s Republic of China

**Keywords:** Polyethylenimine, Chlorotoxin, Drug delivery, SPECT imaging, Glioma

## Abstract

**Background:**

Glioma is the deadliest brain cancer in adults because the blood–brain-barrier (BBB) prevents the vast majority of therapeutic drugs from entering into the central nervous system. The development of BBB-penetrating drug delivery systems for glioma therapy still remains a great challenge. In this study, we aimed to design and develop a theranostic nanocomplex with enhanced BBB penetrability and tumor-targeting efficiency for glioma single-photon emission computed tomography (SPECT) imaging and anticancer drug delivery.

**Results:**

This multifunctional nanocomplex was manufactured using branched polyethylenimine (PEI) as a template to sequentially conjugate with methoxypolyethylene glycol (*m*PEG), glioma-targeting peptide chlorotoxin (CTX), and diethylenetriaminepentaacetic acid (DTPA) for ^99m^Tc radiolabeling on the surface of PEI. After the acetylation of the remaining PEI surface amines using acetic anhydride (Ac_2_O), the CTX-modified PEI (*m*PEI-CTX) was utilized as a carrier to load chemotherapeutic drug doxorubicin (DOX) in its interior cavity. The formed *m*PEI-CTX/DOX complex had excellent water dispersibility and released DOX in a sustainable and pH-dependent manner; furthermore, it showed targeting specificity and therapeutic effect of DOX toward glioma cells in vitro and in vivo (a subcutaneous tumor mouse model). Owing to the unique biological properties of CTX, the *m*PEI-CTX/DOX complex was able to cross the BBB and accumulate at the tumor site in an orthotopic rat glioma model. In addition, after efficient radiolabeling of PEI with ^99m^Tc via DTPA, the ^99m^Tc-labeled complex could help to visualize the drug accumulation in tumors of glioma-bearing mice and the drug delivery into the brains of rats through SPECT imaging.

**Conclusions:**

These results indicate the potential of the developed PEI-based nanocomplex in facilitating glioma-targeting SPECT imaging and chemotherapy. 
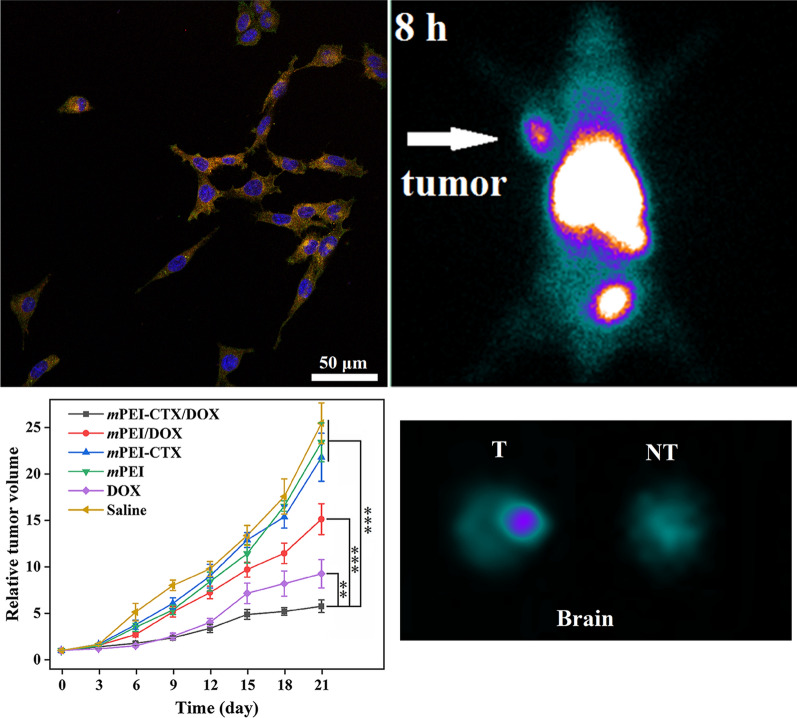

## Background

The diffuse invasion and infiltrative overgrowth of glioma cells lead to the development of irregular and indistinct tumor margins, making surgical resection difficult; consequently, patients with malignant gliomas always have a very poor prognosis [1, 2]. The special pathological and physiological characteristics of the blood–brain barrier (BBB) allow merely a few chemotherapeutic drugs into the brain in the early stage [3]. This strictly limited selection imposes a great challenge on glioma treatment. To overcome this issue, various nanoparticulate platforms have been widely investigated to develop BBB-penetrating delivery systems because of their great clinical potential in cancer diagnosis and treatment [4–6]. These nanoplatforms can be exploited as imaging agents to guide the maximal surgical resection, drug carriers to improve chemotherapy, or theranostic systems to achieve imaging-guided drug delivery and therapy monitoring [7–11].

Although the BBB penetrability of some nanoparticles (NPs) can be improved through specific modification (for example attaching PEG chain or charged/lipophilic groups) [12–14], an active strategy has attracted much more attention [15, 16]. In this active strategy, NPs are generally modified with targeting molecules such as peptides or antibodies, which help NPs bind to endothelial cells of BBB selectively and traverse into the brain via receptor/transporter-mediated transcytosis [17–19]. In previous studies, some receptors and transporters, including but not limited to transferrin receptor, low density lipoprotein receptor, insulin receptor, nicotinic acetylcholine receptor, amino acid transporter, choline transporter, hexose transporter, and monocarboxylic acid transporter, have been determined to be involved in receptor/transporter-mediated transcytosis across BBB [19–26]. These findings have motivated numerous researchers around the world to focus on the development of novel brain targeting systems for glioma imaging and treatment. Moreover, some promising targets, such as the chloride ion channel, which is overexpressed on glioma cells and involved in multiple malignant features of glioma including proliferation, migration, and apoptosis, also arouse intensive interest [27–30]. As a ligand of the chloride ion channel, chlorotoxin (CTX), a small peptide purified from scorpion venom, has been proven to have the ability to penetrate the BBB and show high affinity binding to glioma cells via chloride ion channel and matrix metalloproteinase 2 [31]. Once bound to the receptors on glioma cell surface, the peptide can be internalized into cells. These unique biological properties of CTX make it a potential targeting agent for glioma diagnosis and therapy [32–34]. Perceiving this, the feasibility of using radionuclide ^131^I and fluorescent molecule (indocyanine green) labeled CTX molecules for glioma imaging and treatment has been investigated in clinical trials [35, 36]. Notably, a variety of CTX peptide modified NPs, for instance, CTX-conjugated iron oxides, liposomes, dendrimers, quantum dots, and rare-earth up-conversion NPs, have been studied as potential candidates in this field [37, 38]. These NPs can not only be used as imaging agents for diagnosis or as drug carriers for treatment, but also as multifunctional systems for theranostic applications. Among these NPs, dendritic polymers such as poly(amidoamine) and polyetherimide (PEI) dendrimers, have been considered as promising templates to construct theranostic nanosystems for various kinds of tumors, including brain cancer [39, 40].

In our previous study, we reported CTX-modified dendrimers for glioma imaging and therapy [41]. The developed multifunctional dendrimers exhibited acceptable imaging performance and targeted radionuclide therapy effect, and they could further entrap gold NPs for glioma single-photon emission computed tomography/computed tomography (SPECT/CT) imaging [42]. More importantly, because of the modification of CTX peptide, this kind of dendrimer nanoplatform possesses the ability to cross the BBB and target glioma cells. These results together with the attractive features of CTX facilitated our further investigation of dendrimer-based BBB-penetrating NPs as chemotherapeutic drug carriers for glioma therapy. In addition, compared to the ^131^I used in our previous studies, ^99m^Tc is a better radionuclide for SPECT imaging, which is associated with a higher imaging resolution because of its physical properties such as nearly pure electron capture decay and low γ-ray energy (140 keV) [43, 44]. Therefore, in the present study, a BBB-penetrating imaging-guided drug delivery nanosystem was designed and synthesized using branched PEI as the template by encapsulation of doxorubicin (DOX) and conjugation of CTX peptide and radionuclide ^99m^Tc. DOX was encapsulated into the interior cavities by physical interactions, while CTX and ^99m^Tc were covalently conjugated on the surface of PEI via a PEG chain and bifunctional chelating agent (diethylenetriaminepentaacetic acid, DTPA), respectively. The designed theranostic nanosystem was characterized via different techniques to determine the structure, size, release kinetics, and stability. The targeting specificity and therapeutic efficacy toward glioma cells were evaluated in vitro and in vivo using a subcutaneous glioma tumor model, and the BBB penetrability was investigated via an intracranial rat model, which could be further visualized after the ^99m^Tc radiolabeling through SPECT imaging. Therefore, a combination of glioma-specific agent, chemotherapeutic drug, and radionuclide imaging could be a novel strategy for the imaging-guided drug delivery for brain cancer.

### Methods

#### Synthesis of PEI.NHAc-DOX-(PEG-CTX)-***m***PEG-DTPA-^99m^Tc

**Fig. 1:**
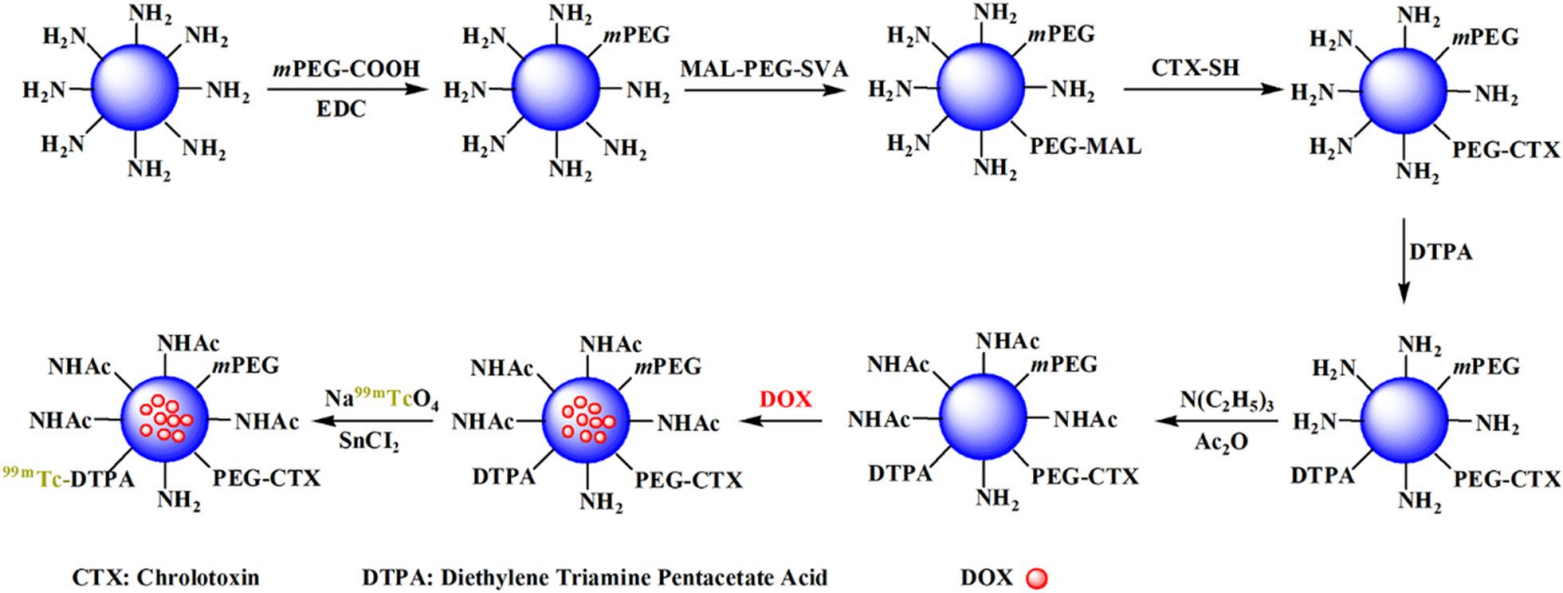
Schematic illustration of the synthesis of the *m*PEI-CTX-^99m^Tc/DOX

In this study, the PEI-based theranostic nanosystem was prepared according to protocols reported in the literature [42]. Briefly, reaction of PEI.NH_2_ with *m*PEG-COOH led to the formation of PEGylated PEI (PEI.NH_2_-*m*PEG). MAL-PEG-SVA was attached to the PEI.NH_2_-*m*PEG to modify with CTX peptide in the next step. To achieve the CTX modification in a convenient way, an extra cysteine residue was added at its C-terminal, which could react with the maleimide group on the PEI surface to form PEI.NH_2_-(PEG-CTX)-*m*PEG. After that, the PEI.NH_2_-(PEG-CTX)-*m*PEG was decorated with DTPA, which is one of the most frequently used bifunctional chelating agent for ^99m^Tc radiolabeling, and the remaining PEI terminal amines were acetylated using excess Ac_2_O. Finally, the PEI.NHAc-DTPA-(PEG-CTX)-*m*PEG (*m*PEI-CTX) was used to encapsulate the anticancer drug DOX, and the synthesized PEI.NHAc-DTPA-(PEG-CTX)-*m*PEG/DOX (*m*PEI-CTX/DOX) complex could be further labeled with ^99m^Tc using SnCl_2_ as the reductant. The schematic illustration of the synthetic process is shown in Fig. 1, and the details are provided in supplementary information.

### Cytocompatibility and cytotoxicity analysis

CCK-8 assay was employed to assess the cytocompatibility of *m*PEI-CTX without DOX and the cell cytotoxicity after DOX encapsulation. For this assay, C6 cells were plated in 96-well plates at a density of 8,000 cells per well and incubated for 24 h. These cells were then treated with *m*PEI-CTX/DOX, *m*PEI/DOX, and free DOX at different DOX concentrations (0–10 μg/mL) or *m*PEI-CTX and *m*PEI at different polymer concentrations (0–100 μg/mL) for 24 h and 48 h, respectively. After washing the cells 3 times with PBS, CCK-8 solution (100 μL) was added to each well, and the cells were cultured for 2 h. The absorbance at 450 nm was measured using a Varioskan Flash multimode microplate reader (Thermo Fisher Scientific, Waltham, MA, USA).

To test the influence of the complex on the cytoskeleton, C6 cells were seeded in a 12-well plate at a density of 2 × 10^5^ cells per well. Subsequently, these cells were incubated with *m*PEI-CTX/DOX, *m*PEI-CTX, and free DOX at the DOX concentration of 5 µg/mL. PBS was set as a negative control. After 24 h, the cytoskeleton was visualized by confocal microscopic imaging using the standard protocols [45].

### Confocal laser scanning microscopy (CLSM) and flow cytometry analysis

Confocal microscopy and flow cytometry analysis were applied to determine the targeting specificity of *m*PEI-CTX/DOX in vitro. For confocal microscopy, C6 cells were seeded into each well of 24-well plates at a density of 5 × 10^4^ and cultured for 24 h. The medium was replaced with 1 mL of fresh medium containing *m*PEI-CTX/DOX or *m*PEI/DOX at the DOX concentration of 8 µg/mL. PBS was set as a negative control. After 4 h, the cells were rinsed, fixed, counterstained, and observed. For flow cytometry analysis, C6 cells were seeded in a 12-well plate (2 × 10^5^ cells per well) and incubated for 24 h. The C6 cells were treated with *m*PEI-CTX/DOX and *m*PEI/DOX at different DOX concentrations (0–8 µg/mL). After 4 h, the cells were harvested and washed 3 times with PBS, and the fluorescence intensities per 10,000 cells were recorded in the FL1-fluorescence channel using a BD AccuriTM C6 Flow Cytometer (BD Biosciences, Franklin Lakes, NJ, USA).

### In vitro SPECT imaging

In vitro SPECT imaging was used to confirm the cellular uptake of *m*PEI-CTX/DOX after ^99m^Tc radiolabeling by glioma cells. In brief, C6 cells were seeded in 12-well plates at a density of 2 × 10^5^ cells/well. After incubation for 24 h, the culture medium was replaced with 2 mL of fresh medium containing *m*PEI-CTX-^99m^Tc/DOX and *m*PEI-^99m^Tc/DOX at different radioactivity concentrations (50, 100, 200, and 400 μCi/mL). After 4 h incubation, the cells were harvested and washed 3 times with PBS, centrifuged in 1.5 mL tubes, and imaged by a SPECT imaging system equipped with Xeleris 2.0 Workstation and low-energy general-purpose collimators (Infinia, Denver, CO).

### Targeted SPECT imaging of glioma in tumor model

Before SPECT imaging in vivo, the mice were divided into targeted and non-targeted groups (6 mice per group) at random. The targeted group was intravenously administrated saline solution containing *m*PEI-CTX-^99m^Tc/DOX (600 μCi, 100 μL), and a same dose of *m*PEI-^99m^Tc/DOX was injected in the non-targeted group. SPECT images were then captured at 0.5, 2, 4, 6, 8, and 12 h post-injection. At 12 h post-injection, one mouse from each group was euthanized, and the tumors and major organs (heart, lung, liver, stomach, spleen, kidneys, soft tissue, and intestines) were collected for analysis of the relative signal intensities.

We subsequently evaluated the therapeutic efficacy of *m*PEI-CTX/DOX in vivo in the subcutaneous tumor model. Ten days after tumor inoculation, the mice were split randomly into 6 groups (6 mice per group). The mice in each group were sequentially treated with *m*PEI-CTX/DOX, *m*PEI/DOX, *m*PEI-CTX, *m*PEI, free DOX, and saline via intravenous injection at a DOX concentration of 1 mg/mL in 100 μL saline solution. The treatments were then performed every 3 days, accounting for a total of 7 times, and the tumor size and body weight of each mouse were recorded after each treatment. Their relative tumor volumes, body weights, and survival rates were calculated as described in our previous work [45]. After a three-week treatment, the representative mice from these groups were sacrificed to harvest the tumors and major organs including the heart, liver, spleen, lung, and kidneys. The hematoxylin and eosin (HE) and terminal deoxynucleotidyl transferase dUTP nick end labeling (TUNEL) assay were performed according to the standard protocols to investigate the potential toxicity of *m*PEI-CTX/DOX in vivo [45]*.*

### Targeted SPECT imaging of glioma in an orthotopic glioma rat model

To verify the BBB penetrability and tumor-targeting efficiency of *m*PEI-CTX/DOX, an orthotopic glioma rat model was established according to the method described previously [42]. Fourteen days later, twelve rats were equally divided into two groups and injected with *m*PEI-CTX-^99m^Tc/DOX and *m*PEI-^99m^Tc/DOX according to the assigned group. SPECT imaging was carried out at 0.5, 2, 4, 6, 8, and 12 h post-injection. All the rats were euthanized after SPECT imaging to separate the brains and measure their relative radioactivity intensities.

### Statistical analysis

Data are presented as mean ± standard deviation, and one-way analysis of variance was performed to evaluate the significance of the data. A p value of 0.05 was selected as the threshold of significance, and the data were denoted with (**) for p < 0.01 and (***) for p < 0.001.

### Results

#### Characterization of the ***m***PEI-CTX-^99m^Tc/DOX complex

The theranostic complexes and the intermediate products obtained during the preparation process were characterized using different techniques. Firstly, ^1^H nuclear magnetic resonance (NMR) spectroscopy was used to characterize the intermediate products including PEI.NH_2_-*m*PEG, PEI.NH_2_-(PEG-MAL)-*m*PEG, PEI.NH_2_-(PEG-CTX)-*m*PEG, PEI.NH_2_-DTPA-(PEG-MAL)-*m*PEG, and PEI.NH_2_-DTPA-(PEG-CTX)-*m*PEG. As shown in Additional file 1: Fig. S1a and S1b, the peaks at 3.5–3.7 ppm could be assigned to the aromatic protons of PEG, while the peaks at 1.0–1.5 ppm were attributed to the protons of CTX (Fig. 1c). By NMR integration analysis, each PEI was estimated to have 13.3 *m*PEG, 14.2 PEG-MAL, and 5.4 CTX moieties. Likewise, the average number of DTPA moieties (at 3.8 ppm) attached onto each PEI was measured to be approximately 7.5 and 7.2, as shown in Additional file 1: Fig. S1d and S1e, respectively.

**Fig. 2:**
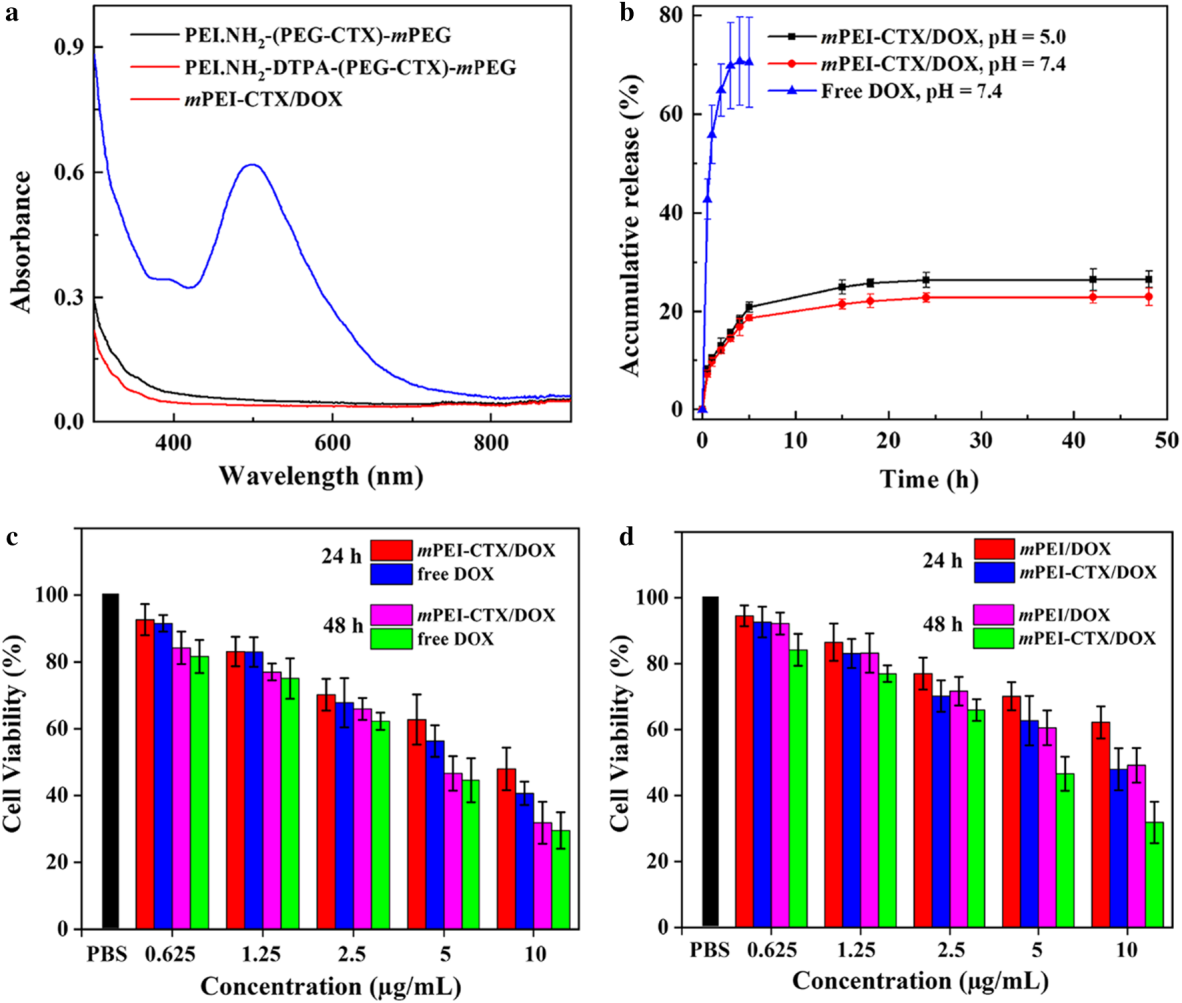
**a** UV–Vis spectra of PEI.NH_2_-(PEG-CTX)-*m*PEG, PEI.NH_2_-DTPA-(PEG-CTX)-*m*PEG, and *m*PEI-CTX/DOX dispersed in water at a polymer concentration of 200 μg/mL. **b** Cumulative release of DOX from *m*PEI-CTX/DOX in PBS buffer (pH = 7.4) and acetate buffer (pH = 5.0) at 37 °C. **c** and **d** CCK-8 assay of C6 cells treated with *m*PEI-CTX/DOX, *m*PEI/DOX, and free DOX at different DOX concentrations for 24 and 48 h

Secondly, after acetylation of the PEI.NH_2_-DTPA-(PEG-CTX)-*m*PEG and PEI.NH_2_-DTPA-(PEG-MAL)-*m*PEG, the *m*PEG-CTX and *m*PEG were formed, and then was used for the encapsulation of DOX to synthesize the *m*PEI-CTX/DOX and *m*PEI/DOX complexes, which could be easily dissolved in different solvents such as water and cell culture media (Additional file 1: Fig. S2a-f). Subsequently, UV–vis spectroscopy was performed to confirm the loading of DOX. As shown in Fig. 2a, the *m*PEI-CTX/DOX complex showed an enhanced absorption at 490 nm, which was related to the typical absorption peak of DOX in the UV–vis spectra, while no absorption at this wavelength could be observed for the intermediate products PEI.NH_2_-(PEG-CTX)-*m*PEG and PEI.NH_2_-DTPA-(PEG-CTX)-*m*PEG without DOX. The amount of DOX loaded within *m*PEI-CTX/DOX complex was calculated to be 20.07 DOX molecules per PEI and the DOX percentage reached 7.02%, which was calculated and analyzed via the standard DOX absorbance/concentration calibration curve (Additional file 1: Fig. S2g-i). Similar results were found for the *m*PEI/DOX complex. Into each PEI, 19.70 DOX molecules were encapsulated, and the DOX percentage was calculated to be 6.99%. Meanwhile, the hydrodynamic size and zeta potential value of *m*PEI-CTX/DOX complex were measured by dynamic light scattering. As shown in Additional file 1: Table S1 and Fig. S3a-c, the hydrodynamic sizes of both *m*PEI-CTX/DOX and *m*PEI/DOX complexes had relatively uniform distributions and were larger than that of *m*PEI-CTX before DOX loading, which reflected the success of DOX loading. As shown in Table S2, the zeta potentials of PEI.NH_2_-DTPA-(PEG-CTX)-*m*PEG and *m*PEI-CTX/DOX showed no significant difference under different pH values, suggesting that the DOX loading did not obviously change the surface potentials of the complexes. Furthermore, the surface potentials of both *m*PEI/DOX and *m*PEI-CTX/DOX complexes under a slightly acidic environment (pH = 5.0) were more positive than those under physiological condition (pH = 7.4). This was likely due to the protonation of partial PEI tertiary amines under a slightly acidic environment (pH = 5.0), as observed in previous studies [46–48].

Thirdly, the release kinetics of DOX from the *m*PEI-CTX/DOX complex were analyzed under two different pH conditions (Fig. 2b). We found that DOX release occurred more rapidly in the initial phase than in the latter, which is in good agreement with that reported previously [45]. At 48 h, the DOX release percentage could be achieved at 26.6% (pH = 5.0) and 22.9% (pH = 7.4), respectively.

Finally, *m*PEI-CTX/DOX was effectively radiolabeled with ^99m^Tc via DTPA. Instant thin-layer chromatography (ITLC) was used to assess the radiochemical yields (RCYs) and stabilities of the ^99m^Tc-labeled PEI-based NPs. The RCYs of *m*PEI-CTX-^99m^Tc/DOX and *m*PEI-^99m^Tc/DOX were found to be 80.3 ± 2.8% and 78.8 ± 0.9% (n = 5), respectively. After PD-10 column purification, the radiochemical purities of both *m*PEI-CTX-^99m^Tc/DOX and *m*PEI-^99m^Tc/DOX were over 99% (Additional file 1: Fig. S3d-f), and remained above 95% after 12 h in phosphate buffered saline (PBS) at room temperature (Additional file 1: Fig. S4a), indicating excellent stabilities in vitro.

### In vitro cytotoxicity assays

CCK-8 assay was used to test the cytocompatibility of *m*PEI-CTX without DOX encapsulation and evaluate the therapeutic efficacy of the *m*PEI-CTX/DOX complex against C6 cells in vitro. As shown in Additional file 1: Fig. S4b, *m*PEI and *m*PEI-CTX displayed little cytotoxicity, and the viabilities of C6 cells after treatment remained more than 90% for all the studied polymer concentrations at 24 h and 48 h. On the contrary, the growth of C6 cells was significantly inhibited by the *m*PEI-CTX/DOX complex and free DOX in a dose-dependent and time-dependent manner (Fig. 2c). After exposure to the *m*PEI-CTX/DOX complex and free DOX for 48 h at the DOX concentration of 10 µg/mL, 31.9% and 29.5% of C6 cells, respectively, survived. The half maximal inhibitory concentration (IC_50_) values of *m*PEI-CTX/DOX and free DOX were calculated to be 9.18 µg/mL and 6.59 µg/mL at 24 h, respectively, and their corresponding IC_50_ values decreased to 4.87 µg/mL and 4.36 µg/mL, respectively, as the incubation time increased to 48 h.

The targeted antitumor efficacy of the *m*PEI-CTX/DOX complex was also evaluated using CCK-8 assay in vitro. Compared with the *m*PEI/DOX complex without CTX modification, the targeted *m*PEI-CTX/DOX complex displayed a stronger inhibitory effect on C6 cells proliferation (Fig. 2d). The viabilities of C6 cells incubated with the *m*PEI-CTX/DOX complex were much weaker than that of the cells treated with *m*PEI/DOX at the same DOX concentrations and time points. The cell survival rate after the *m*PEI-CTX/DOX complex treatment at the DOX concentration of 10 µg/mL for 48 h (31.9%) was much smaller than that after *m*PEI/DOX complex (49.2%) treatment under the same condition.

**Fig. 3:**
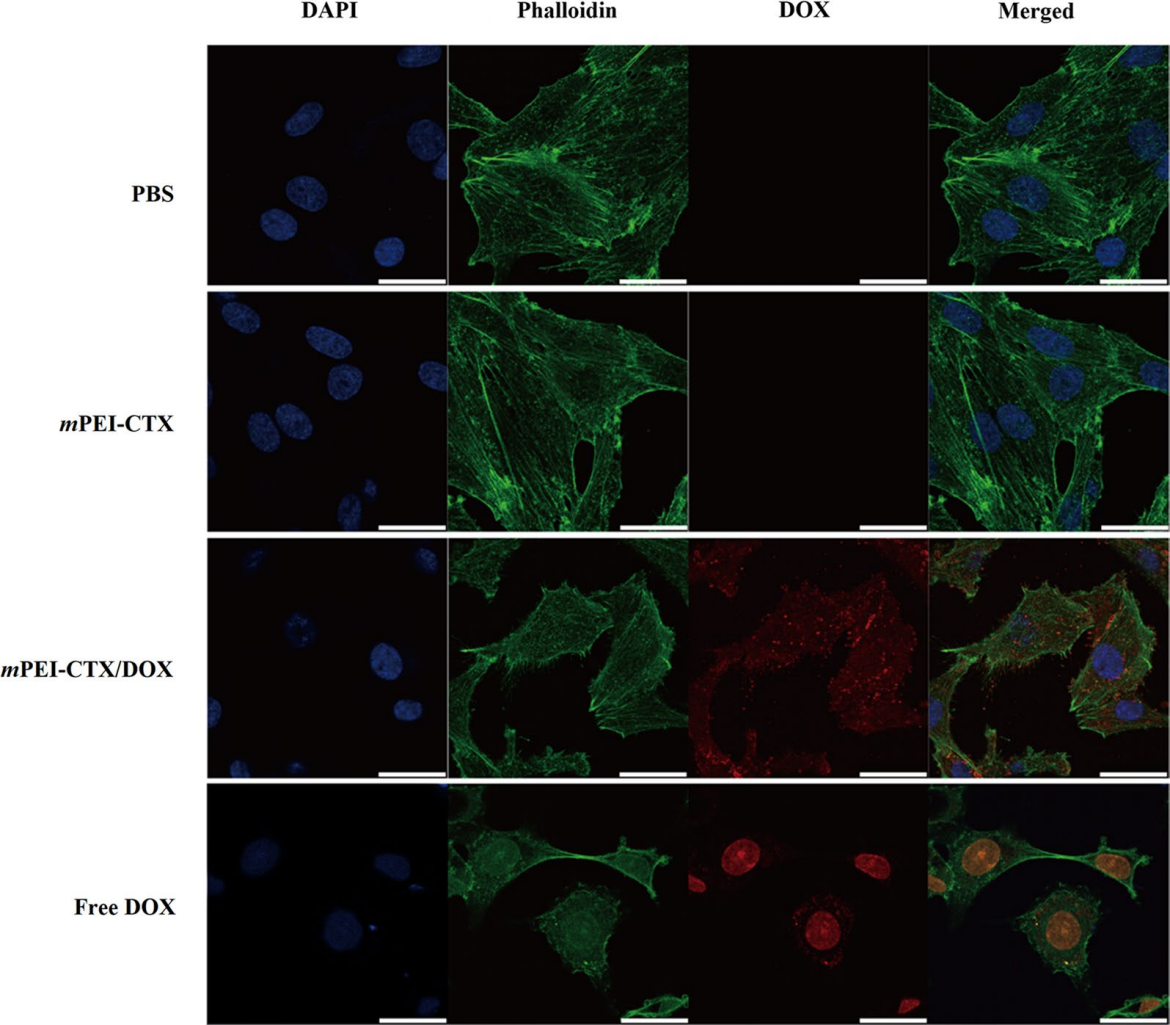
Confocal microscopic images of C6 cells treated with *m*PEI-CTX/DOX, *m*PEI-CTX, free DOX, and PBS for 24 h. The DOX concentration used was 5 μg/mL

Furthermore, we checked the cytoskeleton and nucleus of the cells after treatment (Fig. 3). Obviously, in the absence of DOX such as in the PBS and *m*PEI-CTX groups, the cytoskeleton and nucleus of the treated cells maintained a normal state, and no cytoskeletal injury or cellular membrane dysfunction could be observed. In contrast, severe cytoskeleton damage occurred in C6 cells after incubation with the *m*PEI-CTX/DOX complex and free DOX, and the cytoskeleton of cells treated with the *m*PEI-CTX/DOX complex was almost completely destroyed.

### In vitro targeting specificity

**Fig. 4:**
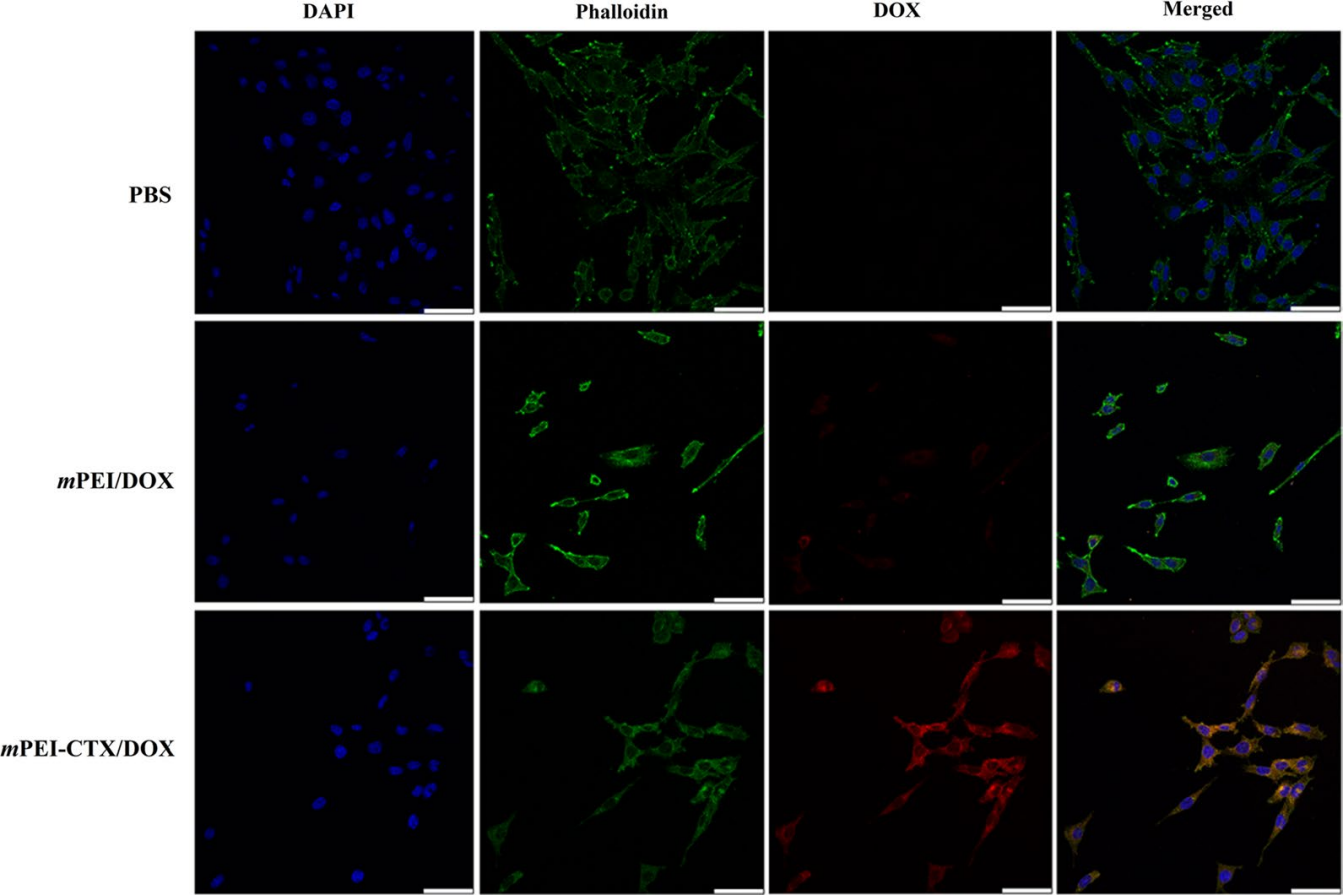
Confocal microscopic images of C6 cells treated with the *m*PEI-CTX/DOX and *m*PEI/DOX at the DOX concentration of 8 μg/mL for 4 h. PBS was used as a control

**Fig. 5:**
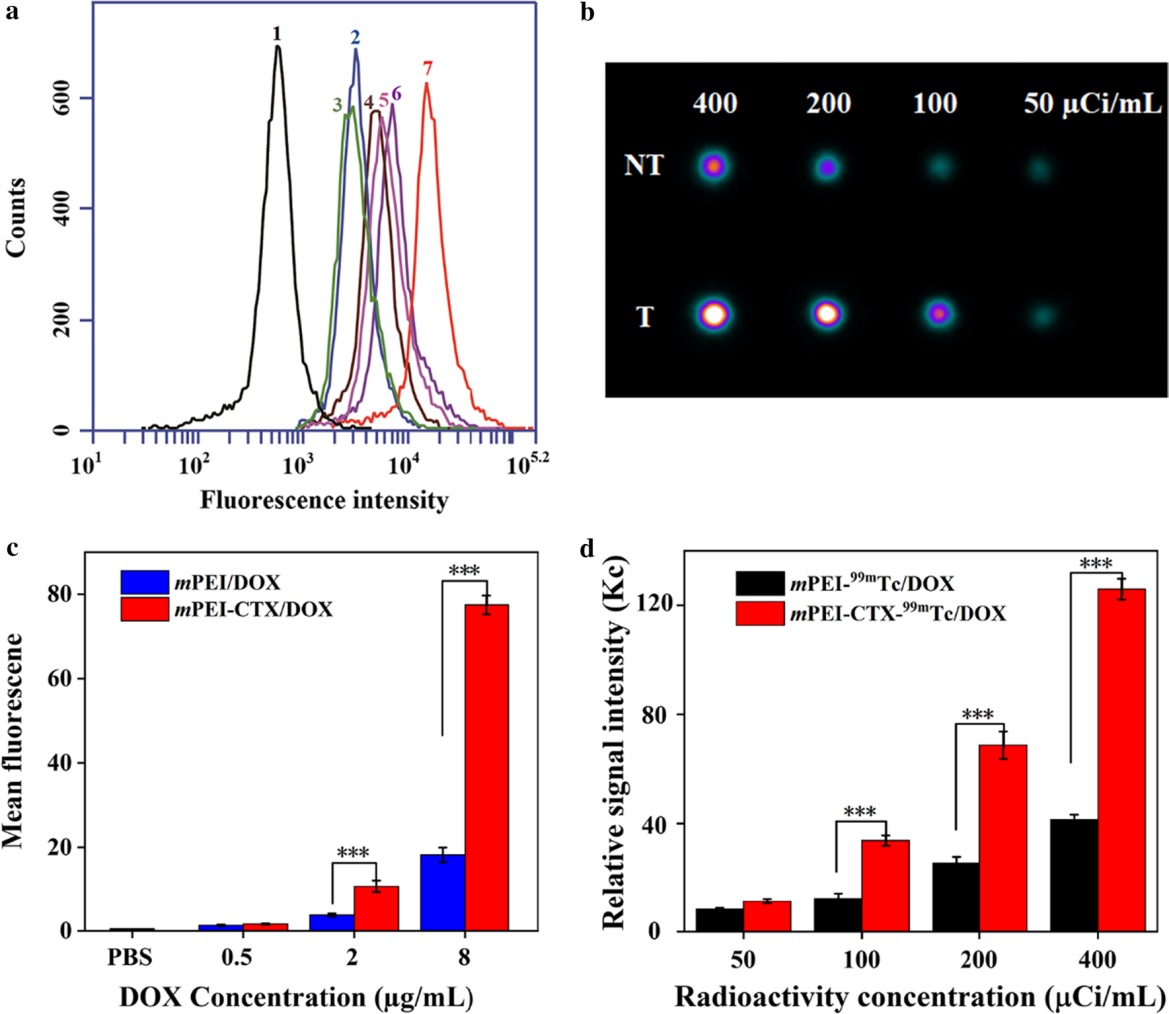
**a** The comparison and **b** flow cytometric analysis of C6 cells treated with *m*PEI-CTX/DOX and *m*PEI/DOX at different DOX concentrations for 4 h. Concentrations were (1) PBS, (2) 0.5 μg/mL *m*PEI/DOX, (3) 0.5 μg/mL *m*PEI-CTX/DOX, (4) 2 μg/mL *m*PEI/DOX, (5) 2 μg/mL *m*PEI-CTX/DOX, (6) 8 μg/mL *m*PEI/DOX, and (7) 8 μg/mL *m*PEI-CTX/DOX. **(c)** In vitro SPECT images of C6 cells treated with *m*PEI-CTX-^99m^Tc /DOX (T) and *m*PEI-^99m^Tc/DOX (NT) for 4 h at the radioactivity concentrations of 50, 100, 200, and 400 μCi/mL, and **d** their relative SPECT signal intensities

To investigate the targeting specificity of the *m*PEI-CTX/DOX complex in vitro, the fluorescence intensities of DOX in C6 cells were qualitatively tested using confocal laser scanning microscopy (CLSM) and quantitatively analyzed using flow cytometry. As shown in Fig. 4, CLSM revealed that the C6 cells incubated with the *m*PEI-CTX/DOX complex had more intense red DOX fluorescence signals both inside the cytosol and on the surface of the cells than those incubated with the *m*PEI/DOX complex. Similarly, because of the presence of DOX, the targeting specificity of the *m*PEI-CTX/DOX complex could be evaluated by flow cytometry. As shown in Fig. 5a and 5b, the C6 cells incubated with the *m*PEI-CTX/DOX complex for 4 h showed significantly higher DOX fluorescence signals compared with those treated with free DOX at the same DOX concentration.

The cellular uptake of *m*PEI-CTX/DOX by C6 cells after ^99m^Tc radiolabeling was also validated in vitro. After incubation with *m*PEI-CTX-^99m^Tc/DOX or *m*PEI-^99m^Tc/DOX for 4 h, SPECT images of these cells were acquired (Fig. 5c and 5d). It could be clearly seen that the cells treated with *m*PEI-CTX-^99m^Tc/DOX displayed higher signal intensities than those treated with *m*PEI-^99m^Tc/DOX at different radioactive concentrations. The SPECT signal intensity of *m*PEI-CTX-^99m^Tc/DOX was much higher than that of *m*PEI-^99m^Tc/DOX at the highest ^99m^Tc concentration.

### In vivo SPECT imaging and antitumor efficacy in a subcutaneous glioma tumor model

**Fig. 6:**
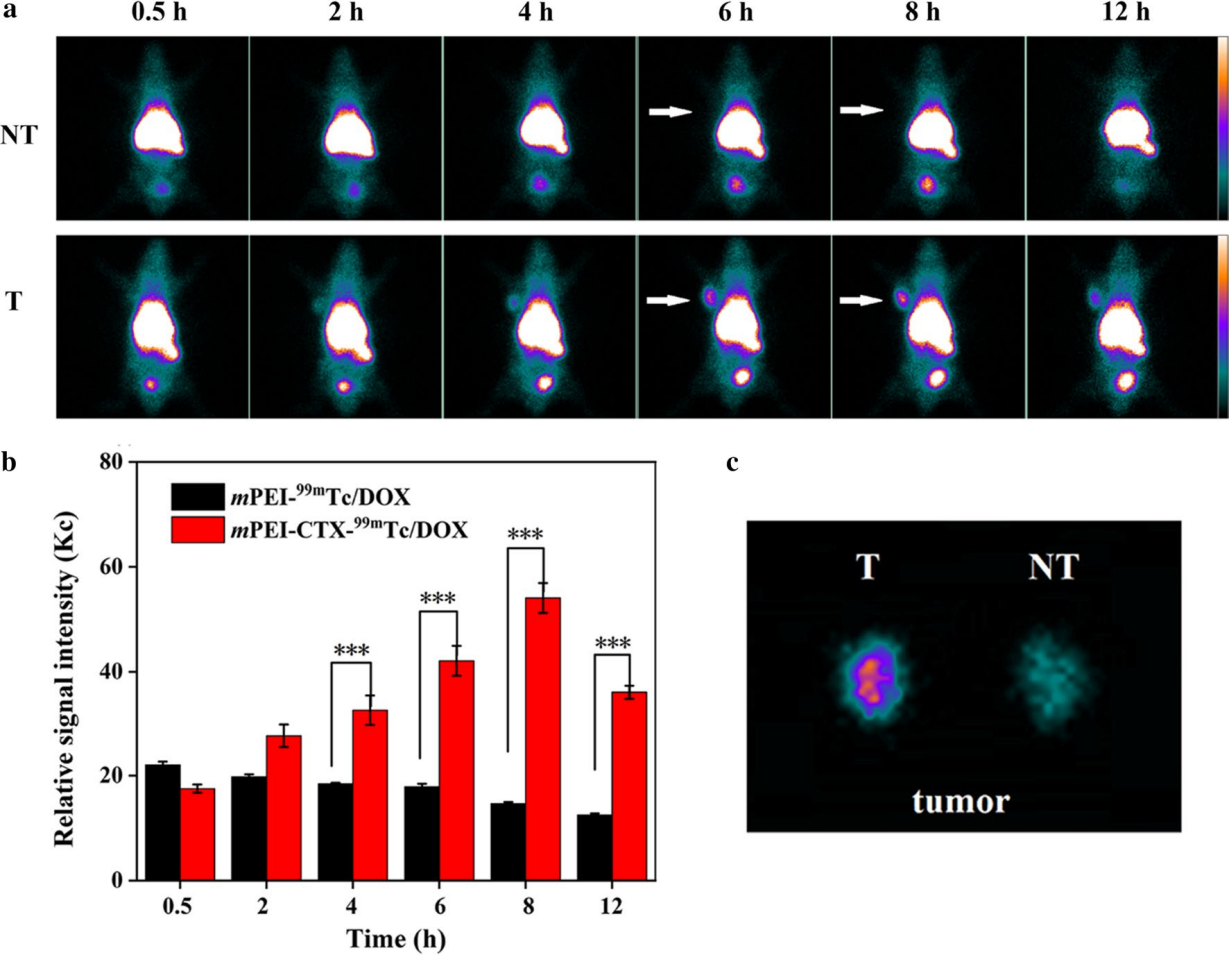
**a** In vivo SPECT images of the nude mice bearing C6 xenografted tumors treated with *m*PEI-CTX-^99m^Tc/DOX (T) and *m*PEI-^99m^Tc/DOX (NT) at different time points of 0.5, 2, 4, 6, 8, and 12 h, respectively, and **b** their tumor relative signal intensities. The white arrows point to the tumor sites. **c** SPECT images of ex vivo tumors at 12 h post-injection

To evaluate the performance of *m*PEI-CTX-^99m^Tc/DOX in vivo, SPECT imaging was performed using a xenografted nude mouse model. Unsurprisingly, the ^99m^Tc-radiolabeled CTX-modified PEI complex exhibited acceptable SPECT imaging results (Fig. 6a and 6b). The tumor accumulation of *m*PEI-CTX-^99m^Tc/DOX could be observable at 2 h post-injection, which increased with the progression of time. Higher signal intensities could be found in tumor regions at 4 h, and 6 h post-injection, and the highest seemed to be at 8 h post-injection followed by attenuated tumor accumulation at 12 h post-injection. Conversely, inconspicuous SPECT signal intensity changes could be found in the tumors for 12 h following the injection of *m*PEI-^99m^Tc/DOX, suggesting the key role of CTX peptide in the process of glioma-targeting. This could be further confirmed by the SPECT image of ex vivo tumors at 12 h post-injection (Fig. 6c), and much higher tumor SPECT signal intensity was observed in the mice treated with *m*PEI-CTX-^99m^Tc/DOX. In addition, biodistribution studies were performed at 12 h post-injection to analyze the accumulation of *m*PEI-CTX-^99m^Tc/DOX and *m*PEI-^99m^Tc/DOX in major organs (Fig. S5). Similar to the high radioactive intensities in SPECT images of the abdomen of mice, the biodistribution data showed that both the *m*PEI-CTX-^99m^Tc/DOX and *m*PEI-^99m^Tc/DOX were mainly accumulated in the liver, kidneys, and spleen with mild accumulation in the lung, heart, and intestines, which resulted in low radioactivity uptake in other orangs such as the stomach and muscle. Notably, the mice treated with *m*PEI-CTX-^99m^Tc/DOX exhibited a higher tumor uptake than those treated with *m*PEI-^99m^Tc/DOX (4.72 ± 0.19 ID%/g *vs* 1.61 ± 0.18 ID%/g), further corroborating the targeting specificity of *m*PEI-CTX-^99m^Tc/DOX in vivo.

**Fig. 7:**
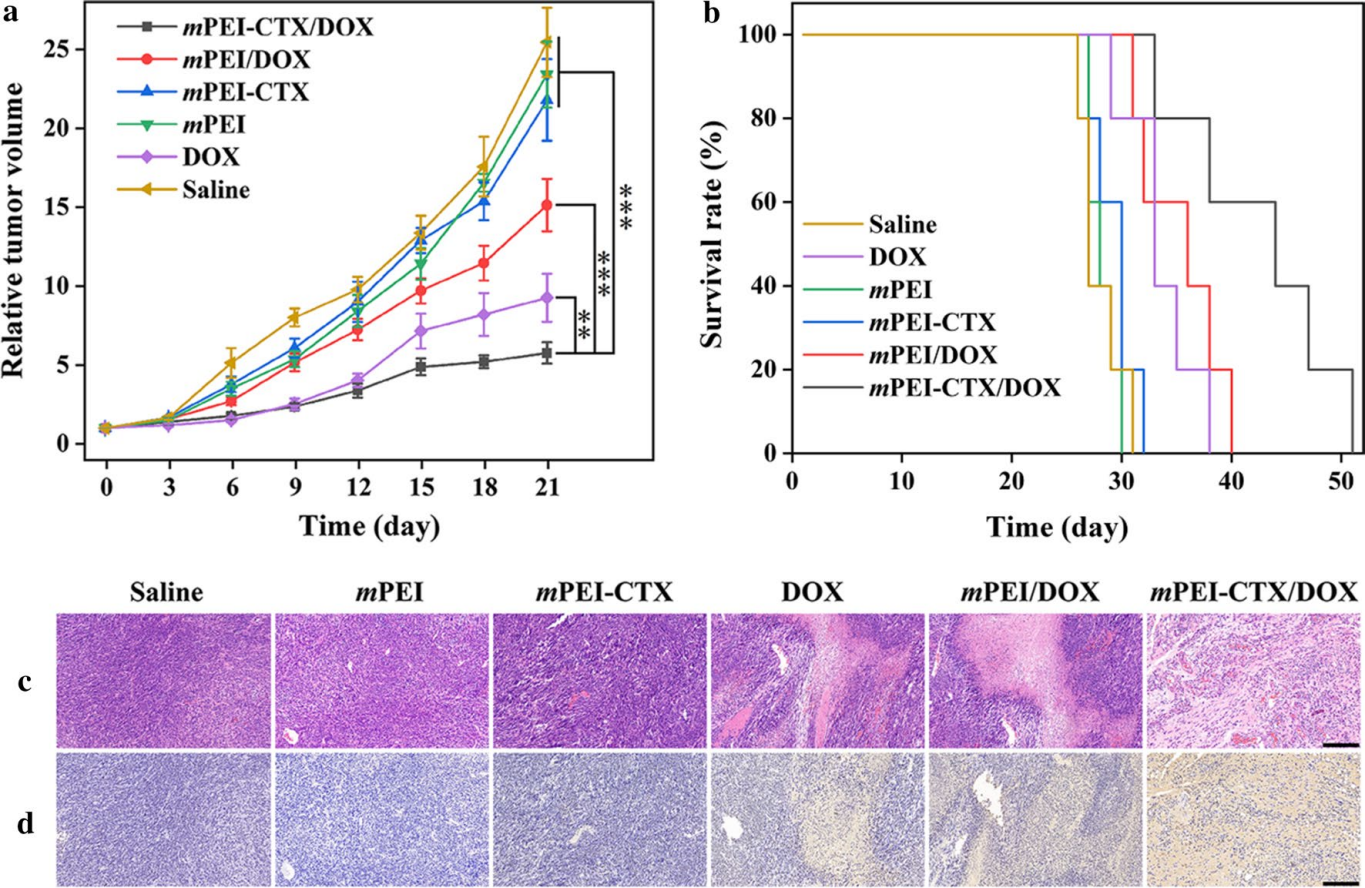
**a** Relative tumor volume of C6 xenografted tumors in nude mice treated with *m*PEI-CTX/DOX, *m*PEI/DOX, *m*PEI-CTX, *m*PEI, DOX, and saline, respectively. The relative tumor volume was normalized according to their initial tumor volume (mean ± SD, n = 6). **b** Survival rate, **c** H&E staining and **d** TUNEL assay results of C6 tumor-bearing mice after various treatments (mean ± SD, n = 6). The scale bar shown in both panels represents 200 μm

Subsequently, the in vivo antitumor effect of the *m*PEI-CTX/DOX complex was investigated using the xenografted tumor model. The *m*PEI/DOX, *m*PEI-CTX, *m*PEI, free DOX, and saline were used as the control groups. The ability to inhibit tumor growth was in the followed the order: *m*PEI-CTX/DOX > free DOX > *m*PEI/DOX > *m*PEI-CTX ≈ *m*PEI ≈ saline (Fig. 7a). Inhibition of tumor growth in the *m*PEI-CTX/DOX complex group was higher than that in the control groups, and the relative tumor volumes after the 21-day treatment in each group had increased 5.77 ± 0.68 (*m*PEI-CTX/DOX), 9.26 ± 1.51 (free DOX), 15.1 ± 1.67 (*m*PEI/DOX), 21.8 ± 2.58 (*m*PEI-CTX), 23.42 ± 2.09 (*m*PEI), and 25.47 ± 2.19 (saline) times, respectively. The antitumor effect of the designed *m*PEI-CTX/DOX complex could also be confirmed by the survival rate data (Fig. 7b). In the studied time period, the survival rate followed the same order of the ability to inhibit tumor growth, and the mice in the *m*PEI-CTX/DOX complex group displayed the longest survival time. The overall survival time was 51 days in the *m*PEI-CTX/DOX complex group and 40 days in the *m*PEI/DOX complex group, which was longer than that in the other control groups. This further demonstrated that the CTX modification enhanced the anti-glioma effect and prolonged survival time by specific targeting. Furthermore, the toxicity and side effects of the drug delivery systems were evaluated according to body weights of the mice during the entire treatment period. As shown in Additional file 1: Fig. S6, a slighter body weight loss was observed in the mice of the free DOX group than those of the other groups, indicating certain toxicity of free DOX to the mice. However, the mice of the *m*PEI-CTX/DOX complex group and those of the other control groups showed no significant differences in weights. This seems to be explained by the inhibited toxicity of DOX after being encapsulated into the PEGylated PEI.

Subsequently, we performed HE and TUNEL staining to check the biosafety and therapeutic effect of the developed *m*PEI-CTX/DOX complex. As shown in Fig. 7c, the H&E staining showed that the tumor sections exhibited well-shaped cells. No obvious necrotic areas could be observed in the *m*PEI-CTX, *m*PEI, and saline groups, while the tumor necrosis was apparent in the other groups. The *m*PEI-CTX/DOX group showed a much larger necrotic area than the *m*PEI/DOX and free DOX groups, indicating that the CTX-modified complex had the strongest anticancer efficiency among the studied groups. Similarly, as shown in Fig. 7d, TUNEL assay revealed no apoptotic cells in the saline, *m*PEI, and *m*PEI-CTX groups. Unlike the *m*PEI/DOX and free DOX groups, which showed a small number of apoptotic cells, the *m*PEI-CTX/DOX group displayed obvious positive staining of apoptotic cells, confirming the antitumor performance in vivo. In addition, the biosafety in the complex in vivo system was checked by observing the H&E stained morphology of the major organs of tumor-bearing mice after treatment (Additional file 1: Fig. S7). Myocardial damage could be found in the free DOX group because of the compound’s cardiotoxicity; however, no obvious damages to the hearts were observed after the encapsulation of DOX into the carriers. As for other major organs, no obvious tissue damage, necrotic areas, or abnormalities could be found in the six groups after treatment. These results revealed the good organ compatibility and low systemic toxicity of the synthesized *m*PEI-CTX/DOX complex to the mice.

### Targeted SPECT imaging of glioma in an orthotopic rat glioma model

**Fig. 8:**
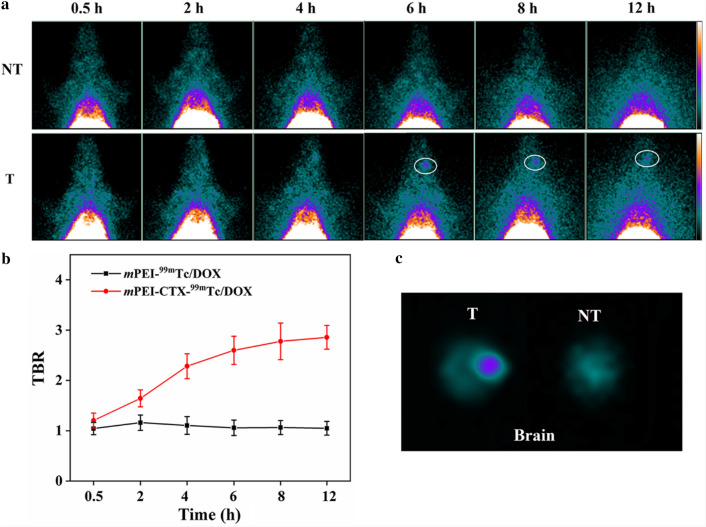
**a** In vivo SPECT images of the rat intracranial glioma model treated with *m*PEI-CTX-^99m^Tc/DOX (T) and *m*PEI-^99m^Tc/DOX (NT) at different time points of 0.5, 2, 4, 6, 8, and 12 h, respectively, and **b** their tumor-to-background ratios (TBR) of the SPECT signal intensities in the brains. **c** SPECT images of ex vivo brains at 12 h post-injection

In view of the unique biological properties of CTX, we evaluated the BBB penetrability and targeting ability of the *m*PEI-CTX/DOX complex using Sprague Dawley (SD) rats bearing intracranial glioma in vivo. The *m*PEI-CTX/DOX and *m*PEI/DOX complexes after ^99m^Tc radiolabeling were injected via tail vein, and their SPECT images were acquired at different time points. As shown in Fig. 8a, the *m*PEI-CTX-^99m^Tc/DOX crossed the BBB, and the tumor uptake in the brains was observable after its accumulation for 2 h, followed by increased signal intensities at tumor sites at 4 h post-injection. The tumor SPECT signal intensities seemed to be stronger at 6 and 8 h post-injection, and they could still be detectable at 12 h post-injection. On the contrary, during the studied period, the rats injected with the *m*PEI-^99m^Tc/DOX complex without CTX modification exhibited insignificant radioactivity accumulation in the glioma regions. These data indicated that CTX peptide could promote the BBB penetrability and glioma-targeting efficiency in PEI-based drug delivery systems. Furthermore, unlike the stable tumor-to-background ratio (TBR) in the rats treated with *m*PEI-^99m^Tc/DOX (Fig. 8b), a rising trend of TBR values was observed in the rats injected with *m*PEI-CTX-^99m^Tc/DOX, which revealed the efficient BBB penetrability and targeting effect. This could also be confirmed by the obvious difference of the signal intensities in the brains resected from the rats after SPECT imaging (Fig. 8c).

## Discussion

Malignant glioma remains the deadliest brain tumor in adults. Among the currently available methods including surgery, radiotherapy and chemotherapy, surgical excision is considered as the primary method; however, it is impossible to remove tumor tissue entirely, leading to an extreme high risk of postoperative recurrence. Radiotherapy can prolong the survival time, but its therapeutic effect is limited to glioma cells that are radiation sensitive. Chemotherapy plays an extremely important role in the comprehensive treatment of gliomas to achieve significant therapeutic effects and good prognosis. Unfortunately, the physiological functions of the BBB allow very few chemotherapy options for gliomas at an early stage, and their obvious toxic and side effects can be brutal, leading to difficult decisions for clinicians and patients. Therefore, investigation of effective methods to promote chemotherapy drugs across BBB has become a focus point in the field of glioma treatment.

With the development of nanomedicine, branched PEI has become a powerful nanocarrier to construct drug delivery systems for various tumors due to its biocompatibility and tunable properties. The PEI surface amines are easily modified with PEG for PEGylation, which has been used as an effective strategy to overcome the systemic clearance and improve the pharmacokinetic properties [45, 47]. In our previous studies, PEGylated PEI was developed as multifunctional templates to entrap gold NPs, label radionuclides, or load drugs for tumor imaging and treatment [48–50]. These PEI-based NPs could also be modified with targeting ligands for different tumors, for example, folic acid for cervical carcinoma and arginine-glycine-aspartic acid peptide for hepatic carcinoma [45, 50]. Moreover, we developed PEI-based NPs for glioma radionuclide imaging and therapy. Due to the modification of CTX, the formed nanosystem possessed the ability to cross the BBB and target glioma cells [42]. In this study, CTX-modified PEI was further explored to build a BBB-penetrating nanocarrier for anticancer drug delivery of DOX, and the tumor accumulation of the prepared nanocarrier could be real-time monitored through SPECT imaging after ^99m^Tc radiolabeling. Following a similar synthesis method, PEI was consecutively connected with *m*PEG, CTX, and DTPA. After acetylation of the remaining PEI surface amines using AcO_2_, *m*PEI-CTX was loaded with DOX to synthesize the *m*PEI-CTX/DOX complex that could be further conveniently radiolabeled with ^99m^Tc for SPECT imaging.

Prior to in vitro biological evaluation, the solubilities and DOX release kinetics of synthesized *m*PEI-CTX/DOX complex were well tested. The data indicated that this complex had excellent dispersibility in different solvents (water, cell culture medium, and methanol), and could sustainably release DOX with a faster rate under the pH value of 5.4. The faster DOX release rate under the acidic condition was likely due to high water solubility of most protonated DOX molecules, which leads to the repulsion of the protonated positively charged PEI backbone and enhanced DOX release from the *m*PEI-CTX/DOX complex. With respect to the anticancer effect on glioma cells, as we expected, *m*PEI-CTX/DOX was found to observably kill glioma cells in a dose- and time-dependent manner, while *m*PEI-CTX without DOX was non-cytotoxic to glioma cells in the given concentration range in the same time period. Clearly, the therapeutic efficacy of PEI-based NPs was only relevant to the antitumor drug DOX. The *m*PEI-CTX/DOX complex showed a higher IC_50_ value than free DOX, which might be ascribed to the gradual release of antitumor drug from the *m*PEI-CTX/DOX complex, that is, the concentration of the released DOX form the complex was lower than that of free DOX at a given time point. Besides, the severe cytoskeleton damage was observed by confocal microscopy in the glioma cells treated with the *m*PEI-CTX/DOX complex and free DOX, further confirming the equivalent therapeutic effect between them in vitro. It was worth noting that the *m*PEI-CTX/DOX complex displayed a stronger inhibitory effect than *m*PEI/DOX on C6 cells proliferation. This result suggested that the targeting ability of CTX made the *m*PEI-CTX/DOX complex suitable for specific cellular uptake, thus enhancing cytotoxicity. The targeting ability of *m*PEI-CTX/DOX was also observed using CLSM and flow cytometry in vitro. Due to the targeting ability of CTX and internalization of the complex into the cytoplasm of C6 cells, the *m*PEI-CTX/DOX had stronger DOX fluorescence intensity than *m*PEI/DOX at the same DOX concentration. In addition, compared to *m*PEI-^99m^Tc/DOX, higher cellular uptake of *m*PEI-CTX-^99m^Tc/DOX by C6 cells suggested that the targeting ability of *m*PEI-CTX/DOX was unaffected by ^99m^Tc radiolabeling. The imaging performance of *m*PEI-CTX-^99m^Tc/DOX in vitro further supported the flow cytometry and CLSM data, which led to the assessment of the applicability of the synthesized complex for targeted SPECT imaging and chemotherapy of glioma models of mice.

SPECT imaging was further used to confirm the tumor accumulation of *m*PEI-CTX/DOX in vivo. In this study, ^99m^Tc was selected for SPECT imaging because of its latent chemical properties for radiolabeling and appropriate half-life (6.02 h), which is fit with the pharmacokinetic characteristics of the formed complex. In agreement with the findings of our group and other researchers, CTX-modified NPs could selectively bind to glioma cells in the subcutaneous tumor mouse model, and be able to cross the BBB and accumulate at the tumor site in the orthotopic glioma model [32–34, 41, 42]. The tumor accumulation of *m*PEI-CTX-^99m^Tc/DOX could be detected at 2 h post-injection, increase as time prolonged and reach a peak at 8 h post-injection. Even 12 h after injection, the tumor accumulation was not significantly attenuated, which might be attributed to the synergistic effect between PEGylation and targeting ability of CTX. The PEGylation modification prolonged the blood circulation time of the complex, and CTX endowed the complex with target specificity toward glioma cells in vivo, leading to an improved cellular uptake and retention time in tumor region. The good accumulation and long-term retention of chemotherapeutic drug in tumor were indispensable for targeting therapy, as it can maximize the therapeutic effect with minimized toxicity and side effects. Owing to the advantage of PEI-based drug delivery system and the existence of CTX, *m*PEI-CTX/DOX not only could obviously inhibit the subcutaneous tumor growth and prolong the lifetime of the tumor-bearing mice, but also did not cause any significant damage to vital organs such as the heart, liver and kidneys, when compared to the direct treatment with DOX alone. These results highlighted the potential of formed *m*PEI-CTX/DOX complex as an anticancer drug delivery system for glioma treatment; however, additional studies should be conducted to investigate the therapeutic effect of this complex on intracranial gliomas and its value in therapeutic monitoring in future studies.

## Conclusions

In this study, we designed and synthesized a PEI-based drug delivery system for targeted glioma therapy. The dendritic PEI could be readily modified with CTX peptide on the surface, and effectively encapsulate the anticancer drug DOX into the interior cavities. This CTX-functionalized PEI-based drug delivery system could release DOX in a pH-sensitive manner and displayed good targeting ability and therapeutic efficacy toward glioma cells in vitro and in vivo (a subcutaneous tumor model). More importantly, owing to the unique biological characters of CTX, the developed drug delivery system was able to cross BBB and accumulate in the brain tumor region, and the tumor accumulation could be visualized after ^99m^Tc radiolabeling. This PEI-based drug carrier not only exhibited a potential strategy to overcome the challenges posed by the BBB barrier through peptide modification, but also provided a promising approach to fabricate imaging-guided drug delivery systems for different types of cancers.

## Supplementary information

**Additional file 1: Table S1.** Hydrodynamic sizes of PEI.NH_2_-DTPA-(PEG-CTX)-*m*PEG, *m*PEI-CTX/DOX and *m*PEI/DOX complexes dispersed in water. **Table S2.** Zeta potential values of PEI.NH_2_-DTPA-(PEG-CTX)-*m*PEG, *m*PEI-CTX/DOX and *m*PEI/DOX under different pH conditions. **Fig. S1. **^**1**^H NMR spectra of **a** PEI.NH_2_-*m*PEG, **b** PEI.NH_2_-(PEG-MAL)-*m*PEG, **c** PEI.NH_2_-(PEG-CTX)-*m*PEG, **d** PEI.NH_2_-DTPA-(PEG-MAL)-*m*PEG and **e** PEI.NH_2_-DTPA-(PEG-CTX)-*m*PEG, respectively. **f** Schematic illustration of the *m*PEI-CTX structure. **Fig. S2.** Photographs of the *m*PEI-CTX/DOX (**a** and **b**) and *m*PEI/DOX (**d** and **e**) dispersed in water (**a** and **d**), cell culture medium (**b** and **e**), respectively, and blank cell culture medium (**c** and **f**). Standard curve of DOX dissolved in methanol (**g**) and PBS with pH 5.0 (**h**) and 7.4 (**i**). **Fig. S3.** Hydrodynamic size distributions of **a** PEI.NH_2_-DTPA-(PEG-CTX)-*m*PEG, **b** *m*PEI/DOX and **c** *m*PEI-CTX/DOX dispersed in water. ITLC results of **d** Na^99m^TcO_4_, **e** *m*PEI-^99m^Tc/DOX and **f** *m*PEI-CTX-^99m^Tc/DOX on silica gel-coated fiber glass sheets using saline as the mobile phase. **Fig. S4. a** Radiochemical purities of *m*PEI-CTX-^99m^Tc/DOX and *m*PEI-^99m^Tc/DOX in PBS at room temperature and FBS at 37 ºC for 1, 2, 6 and 12 h. **b** CCK-8 assay of C6 cells treated with the *m*PEI or *m*PEI-CTX at different polymer concentrations for 24 and 48 h, respectively. **Fig. S5.** Biodistribution of the *m*PEI-CTX-^99m^Tc/DOX and *m*PEI-^99m^Tc/DOX at 12 h post-injection. **Fig. S6.** Body weight changes of the C6 tumor-bearing mice during the treatments with *m*PEI-CTX/DOX, *m*PEI/DOX, *m*PEI-CTX, *m*PEI, DOX and saline, respectively. The relative body weight was normalized according to their initial weights (Mean ± SD, n = 6). **Fig. S7.** H&E staining of the heart, liver, spleen, lung and kidney of the tumor-bearing mice after the 21-day treatment with *m*PEI-CTX/DOX, *m*PEI/DOX, *m*PEI-CTX, *m*PEI, DOX and saline. The scale bar in each panel indicates 200 μm.

## Data Availability

All data generated or analyzed during this study are included in this published article.

## Abbreviations

ANOVA: Analysis of variance
BBB: Blood–brain-barrier
CLSM: Confocal laser scanning microscopy
CTX: Chlorotoxin
DMEM: Dulbecco's Modified Eagle medium
DMSO: Dimethyl sulfoxide
DTPA: Diethylenetriaminepentaacetic acid
FI: Fluorescein isothiocyanate
H&E: Hematoxylin and eosin
ITLC: Instant thin-layer chromatography
*m*PEG: Methoxypolyethylene glycol
NMR: Nuclear magnetic resonance;
NP: Nanoparticle
PBS: Phosphate buffered saline
PEG: Polyethylene glycol
PEI: Polyethyleneimine
RCY: Radiochemical yield
SPECT: Single-photon emission computerized tomography
TBR: Tumor-to-background ratio
TUNEL: Transferase dUTP nick end labeling
