# Supplementary material for: Polyethylenimine-based theranostic nanoplatform for glioma-targeting single-photon emission computed tomography imaging and anticancer drug delivery
Source: J Nanobiotechnology. 2020 Oct 14;18:143. doi: 10.1186/s12951-020-00705-3 (PMC7557081; doi:10.1186/s12951-020-00705-3)
Supplement: Supplementary file 1 — Additional file 1: Table S1. Hydrodynamic sizes of PEI.NH2-DTPA-(PEG-CTX)-mPEG, mPEI-CTX/DOX and mPEI/DOX complexes dispersed in water. Table S2. Zeta potential values of PEI.NH2-DTPA-(PEG-CTX)-mPEG, mPEI-CTX/DOX and mPEI/DOX under different pH conditions. Fig. S1. 1H NMR spectra of a PEI.NH2-mPEG, b PEI.NH2-(PEG-MAL)-mPEG, c PEI.NH2-(PEG-CTX)-mPEG, d PEI.NH2-DTPA-(PEG-MAL)-mPEG and e PEI.NH2-DTPA-(PEG-CTX)-mPEG, respectively. f Schematic illustration of the mPEI-CTX structure. Fig. S2. Photographs of the mPEI-CTX/DOX (a and b) and mPEI/DOX (d and e) dispersed in water (a and d), cell culture medium (b and e), respectively, and blank cell culture medium (c and f). Standard curve of DOX dissolved in methanol (g) and PBS with pH 5.0 (h) and 7.4 (i). Fig. S3. Hydrodynamic size distributions of a PEI.NH2-DTPA-(PEG-CTX)-mPEG, b mPEI/DOX and c mPEI-CTX/DOX dispersed in water. ITLC results of d Na99mTcO4, e mPEI-99mTc/DOX and f mPEI-CTX-99mTc/DOX on silica gel-coated fiber glass sheets using saline as the mobile phase. Fig. S4. a Radiochemical purities of mPEI-CTX-99mTc/DOX and mPEI-99mTc/DOX in PBS at room temperature and FBS at 37 ºC for 1, 2, 6 and 12 h. b CCK-8 assay of C6 cells treated with the mPEI or mPEI-CTX at different polymer concentrations for 24 and 48 h, respectively. Fig. S5. Biodistribution of the mPEI-CTX-99mTc/DOX and mPEI-99mTc/DOX at 12 h post-injection. Fig. S6. Body weight changes of the C6 tumor-bearing mice during the treatments with mPEI-CTX/DOX, mPEI/DOX, mPEI-CTX, mPEI, DOX and saline, respectively. The relative body weight was normalized according to their initial weights (Mean ± SD, n = 6). Fig. S7. H&E staining of the heart, liver, spleen, lung and kidney of the tumor-bearing mice after the 21-day treatment with mPEI-CTX/DOX, mPEI/DOX, mPEI-CTX, mPEI, DOX and saline. The scale bar in each panel indicates 200 μm. [file 12951_2020_705_MOESM1_ESM.docx]

**Supplementary Information**

**Polyethylenimine-Based Theranostic Nanoplatform for Glioma-Targeting Single-Photon Emission Computed Tomography Imaging and Anticancer Drug Delivery**

**Lingzhou Zhao^1^**^§^**, Jingyi Zhu^2^**^§^**, Jiali Gong^1^**^§^**,** **Ningning Song^1^, Shan Wu^1^, Wenli Qiao^1^,** **Jiqin Yang^3*^, Meilin Zhu^4*^ and Jinhua Zhao^1*^**

^1^ Department of Nuclear Medicine, Shanghai General Hospital, Shanghai Jiao Tong University School of Medicine, Shanghai 200080, People’s Republic of China

^2^School of Pharmaceutical Sciences, Nanjing Tech University, Nanjing 211816, People’s Republic of China

^3^ Department of Nuclear Medicine, General Hospital of Ningxia Medical University, Yinchuan 750004, Ningxia, People’s Republic of China

^4^ School of Basic Medical Sciences, Ningxia Medical University, Yinchuan 750004, Ningxia, People’s Republic of China

________________________________________________________

* Corresponding authors: zhaojinhua1963@126.com (J. Zhao), Fax: 0086-21-37798352, Tel: 0086-21-37798352; [jay70281@163.com](mailto:jay70281@163.com) (M. Zhu); [qin-yj06@163.com](mailto:qin-yj06@163.com) (J. Qin)

^§^ These authors equally contributed to this work.

**Materials**

Branched PEI (PEI-NH_2_, Mw = 25,000), 1-ethyl-3-(3-(dimethylamino)propyl) carbodiimide hydrochloride (EDC), stannous chloride, acetic anhydride (Ac_2_O), dimethyl sulfoxide (DMSO), and triethylamine were supplied by Sigma-Aldrich (St. Louis, MO). PEG monomethyl ether with one end terminating in a carboxyl group (*m*PEG-COOH, Mw = 5,000), Maleimide-PEG-succinimidyl valerate (MAL-PEG-SVA, Mw = 5,000), and cellulose dialysis membranes were obtained from Shanghai Yanyi Biotechnology Corporation (Shanghai, China). The amino acid sequence of CTX peptide is MCMPCFTTDHQMARKCDDCCGGKGRGKCYGPQCLCR with four disulfide bridges (Cys2-Cys19, Cys5-Cys28, Cys16-Cys33 and Cys20-Cys35) and was manufactured by Shanghai Bootech BioScience & Technology Co., Ltd. (Shanghai, China). ^99m^Tc-pertechnetate (Na^99m^TcO_4_) solution was obtained from Shanghai GMS Pharmaceutical Co., Ltd. (Shanghai, China). Diethylenetriaminepentaacetic acid (DTPA), disposable PD-10 desalting columns, DOX, fluorescein isothiocyanate (FI), cell counting kit-8 (CCK-8), phosphate buffered saline (PBS), fetal bovine serum (FBS), RPMI 1640 medium, penicillin, and streptomycin were procured from Shanghai Dobio CO., Ltd. (Shanghai, China). All other chemicals and solvents were supplied by Sinopharm Chemical Reagent Co., Ltd. (Shanghai, China).

**Cell lines and animals**

C6 cells were purchased from Cell Bank of Chinese Academy of Science (Shanghai, China), and were cultured in Dulbecco's Modified Eagle medium (DMEM) supplemented with 10% FBS, 100 U/mL penicillin, and 100 μg/mL streptomycin in 5% CO_2_ at 37 ºC. Female nude mice (18−20 g) were purchased from Shanghai Slac Laboratory Animal Center (Shanghai, China) and subcutaneously injected with 2 × 10^6^ C6 cells in the right-side flank. When the tumor volume reached approximately 0.6–1.0 cm^3^, the mice were ready for experiments.

**Synthesis of *m*PEI-CTX-^99m^Tc/DOX**

Briefly, *m*PEG-COOH (900 mg) was activated by EDC (517.59 mg), and then were mixed with PEI.NH_2_ (300 mg) dissolved in DMSO under constant stirring for 3 days to obtain PEI.NH_2_-*m*PEG. MAL-PEG-SVA (900 mg) dissolved in DMSO was slowly added to the mixture. After another 3 days, CTX-SH (172.65 mg) was reacted with the MAL groups on the PEI surface overnight to synthesize the PEI.NH_2_-(PEG-CTX)-*m*PEG conjugates. DTPA (51.45 mg) was then added to the reaction mixture with stirring overnight to obtain the PEI.NH_2_-DTPA-(PEG-CTX)-*m*PEG. The remaining PEI terminal amines were further acetylated by mixing with triethylamine (5.87 mL) and Ac_2_O (3.32 mL) with stirring for 24 h. After purification using a 14 kDa molecular weight cut-off membrane against PBS (3 times, 2 L) and water (5 times, 2 L) over 3 days to remove the excess reactants and byproducts, the formed PEI.NHAc-DTPA-(PEG-CTX)-*m*PEG (*m*PEI-CTX) were acquired. The intermediates were collected and characterized to assess the average number of conjugated molecules (DTPA, CTX and *m*PEG) per PEI dendrimer.

Subsequently, DOX·HCl (13.12 mg) with 30 molar equiv. of the *m*PEI-CTX was dissolved in 500 µL methanol and neutralized by addition of 20 µL of triethylamine to form DOX. The DOX solution was then added into an aqueous *m*PEI-CTX solution (125.1 mg, 5 mL in water) and the mixture was vigorously stirred overnight to evaporate the methanol solvent. Then the mixture solution was centrifuged (7,000 rpm) for 10 min to collect the precipitate, which is associated with the noncomplexed free DOX. The collected DOX precipitate was then utilized to [quantitative](javascript:;)ly analyze the drug loading efficiency of *m*PEI-CTX/DOX complex *via* UV-vis spectroscopy. The supernatant solution was collected and lyophilized to obtain the *m*PEI-CTX/DOX complex.

Finally, the *m*PEI-CTX/DOX complex was labeled with ^99m^Tc *via* the DTPA ligands. Briefly, Na^99m^TcO_4_ solution (20 mCi, 100 μL) was mixed with stannous chloride (200 μg) and *m*PEI-CTX/DOX complex (200 μg) dissolved in 250 μL of PBS (0.1 M, pH = 7.2–7.4) with continuous stirring. After incubation for 30 min at 37 °C, the reaction mixture was purified using PD-10 desalting columns with saline as the mobile phase, and 1 mL of liquid was collected in each tube. After 10 tubes, the radioactivity of each tube was measured using a CRC-15R radioisotope dose calibrator (Capintec, Inc., Ramsey, NJ). The *m*PEI-CTX-^99m^Tc/DOX complex could be found in the third and fourth tube. The *m*PEI-^99m^Tc/DOX complex without CTX modification was also prepared using the same experimental conditions. Their radiostabilities *in vitro* were assessed by measuring the radiochemical purities at different time intervals using instant thin-layer chromatography (ITLC). The ITLC consisted of silica gel-coated fiber glass sheets (Macherey-Nagel, GmbH & Co. KG, Düren, Germany) and a thin-layer chromatogram scanner installed with γ-counter (Bioscan Inc., Tucson, AZ). Saline was utilized as the mobile phase, and the sheets were analyzed by the scanner.

**Characterization techniques**

^1^H NMR spectra of samples dissolved in D_2_O were obtained using a Bruker AV400 nuclear magnetic resonance spectrometer (Bruker AXS Advanced X-ray Solutions GmbH, Karlsruhe, Germany). UV-vis spectra were measured using a Lambda 25 UV-vis spectrophotometer (PerkinElmer, Inc., Waltham, MA, USA). Hydrodynamic sizes and zeta potentials of all the acquired samples dissolved in water were measured by dynamic light scattering using a Malvern Zetasizer Nano ZS model ZEN 3600 (Malvern Panalytical Ltd., Malvern, UK) with a standard 633 nm laser. ITLC was performed by silica gel-coated fiber glass sheets (Macherey-Nagel, GmbH & Co. KG, Düren, Germany) using saline as the mobile phase. The sheets were analyzed using the thin layer chromatogram scanner (Bioscan Inc., Tucson, AZ). SPECT imaging was performed using a GE Infinia SPECT scanner equipped with an Xeleris workstation and low-energy general-purpose collimators (GE Healthcare).

***In vitro* drug release**

The *m*PEI-CTX/DOX complex (3 mg) was dispersed into 1 mL of PBS (pH 7.4) or acetate buffer (pH 5.0) and placed in a dialysis bag (MWCO = 14 000). The dialysis bag was then immersed in the corresponding buffer medium with a volume of 9 mL and kept in a vapor-bathing constant temperature vibrator at 37 °C. At each time point, 1 mL of outer phase medium was taken out and the same volume of fresh corresponding buffer medium was replenished. As control, the release experiment of free DOX·HCl dissolved in 1 mL of PBS (pH 7.4) was carried out using the same method at the pH of 7.4. The collected outer phase media at different specific time points were quantified by UV-vis spectroscopy.

**Table S1** Hydrodynamic sizes of PEI.NH_2_-DTPA-(PEG-CTX)-*m*PEG, *m*PEI-CTX/DOX, and *m*PEI/DOX complexes dispersed in water

| Materials | Hydrodynamic size (nm) |
| --- | --- |
| PEI.NH_2_-DTPA-(PEG-CTX)-*m*PEG | 204.03 ± 10.23 |
| *m*PEI-CTX/DOX | 394.77 ± 41.25 |
| *m*PEI/DOX | 364.73 ± 25.12 |

**Table S2** Zeta potential values of PEI.NH_2_-DTPA-(PEG-CTX)-*m*PEG, *m*PEI-CTX/DOX, and *m*PEI/DOX complexes under different pH conditions

| Materials | Zeta potenial (mV) | | |
| --- | --- | --- | --- |
|  | pH = 5.0 | pH = 7.4 | pH = 10.0 |
| PEI.NH_2_-DTPA-(PEG-CTX)-(*m*PEG) | 2.55 ± 1.02 | 1.48 ± 0.28 | -1.35 ± 0.47 |
| *m*PEI-CTX/DOX | 4.41 ± 0.43 | 1.17 ± 0.11 | -1.88 ± 0.17 |
| *m*PEI/DOX | 4.23 ± 0.31 | 0.92 ± 0.23 | -1.16 ± 0.48 |


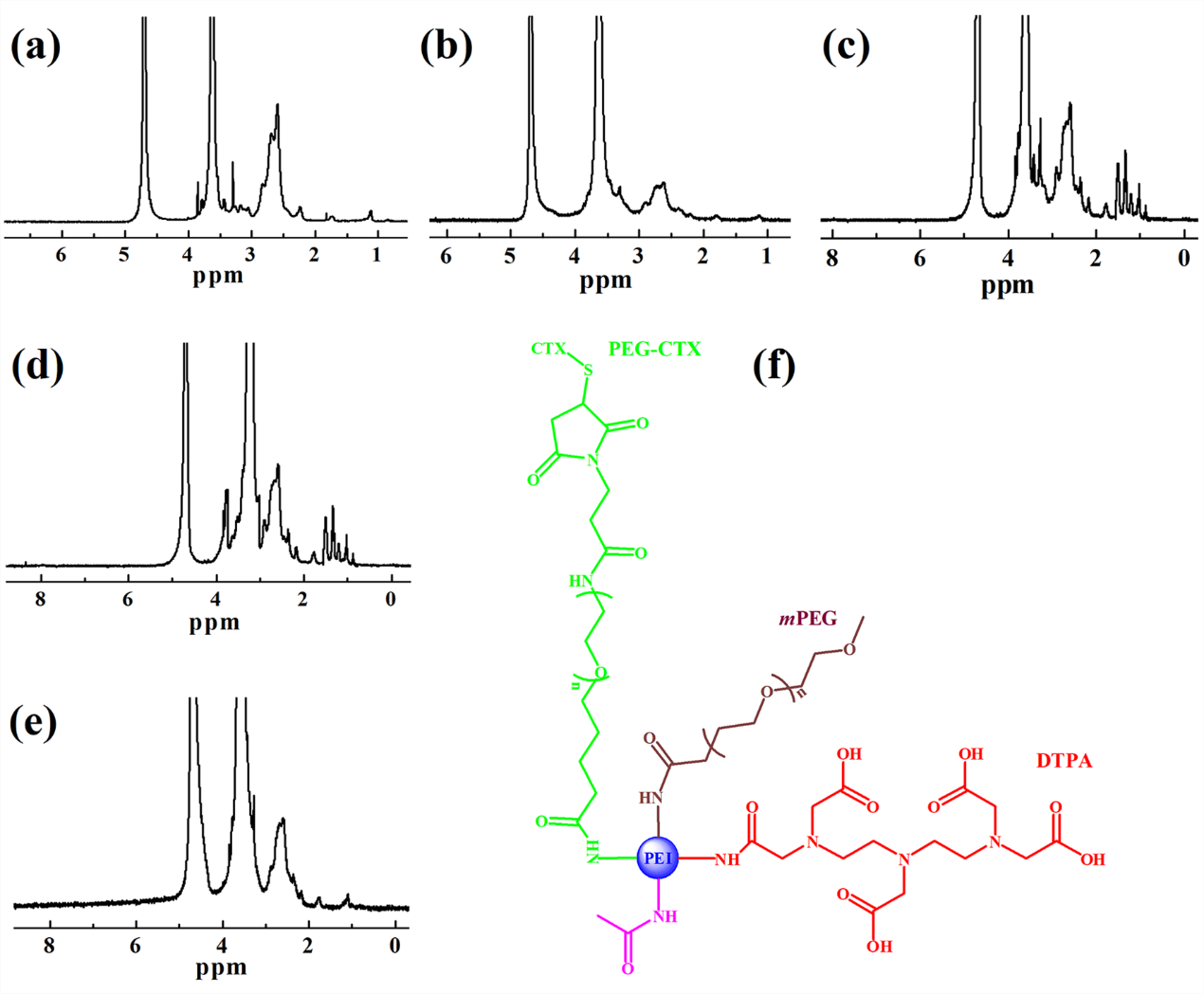


**Fig. S1** **^1^**H NMR spectra of **a** PEI.NH_2_-*m*PEG, **b** PEI.NH_2_-(PEG-MAL)-*m*PEG, **c** PEI.NH_2_-(PEG-CTX)-*m*PEG, **d** PEI.NH_2_-DTPA-(PEG-MAL)-*m*PEG, and **e** PEI.NH_2_-DTPA-(PEG-CTX)-*m*PEG, respectively. **f** Schematic illustration of the *m*PEI-CTX structure.


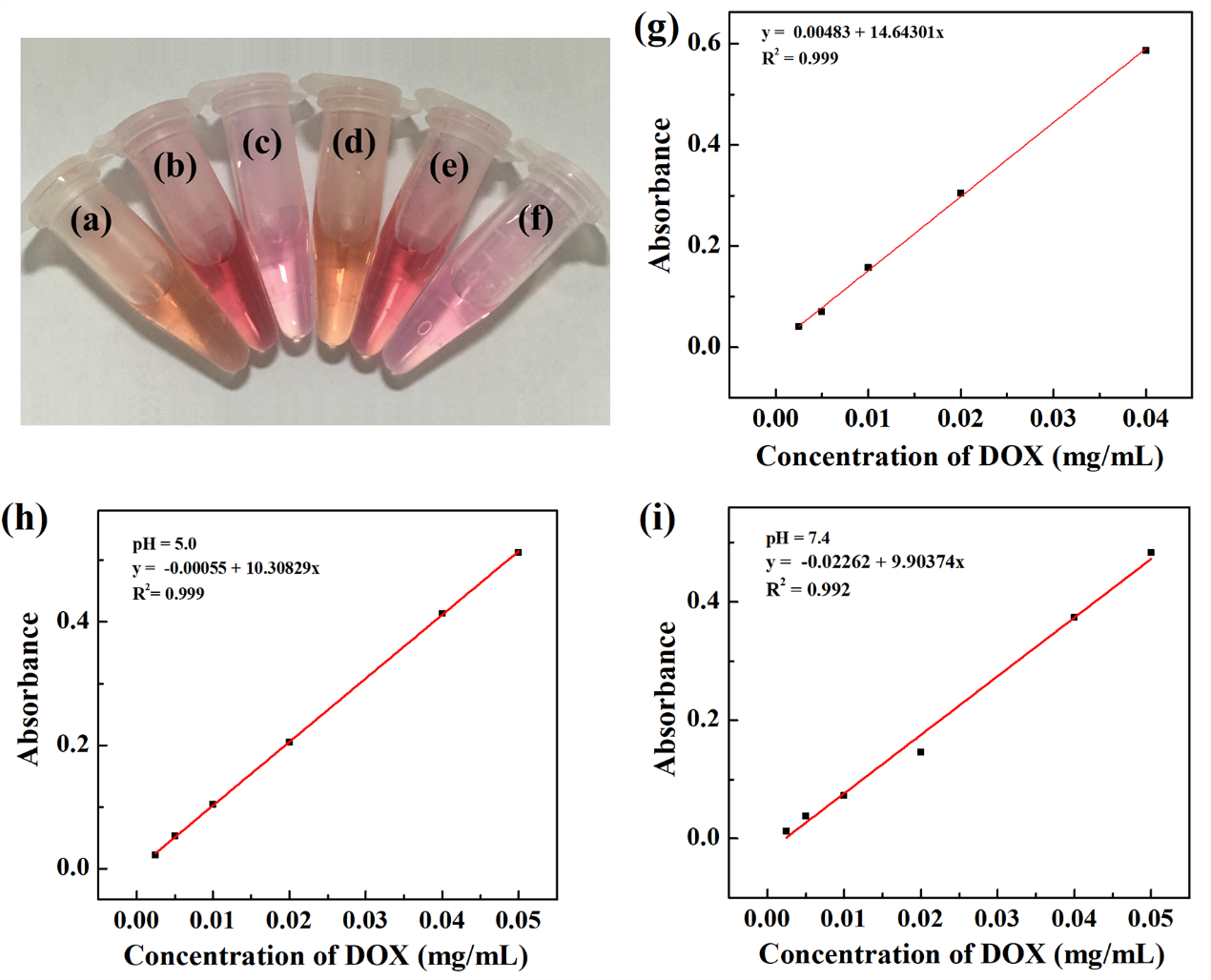


**Fig. S2** Photographs of the *m*PEI-CTX/DOX (**a** and **b**) and *m*PEI/DOX (**d** and **e**) dispersed in water (**a** and **d**) and cell culture medium (**b** and **e**), respectively, and blank cell culture medium (**c** and **f**). Standard curve of DOX dissolved in methanol (**g**) and PBS at pH of 5.0 5.0 (**h**) and 7.4 (**i**).


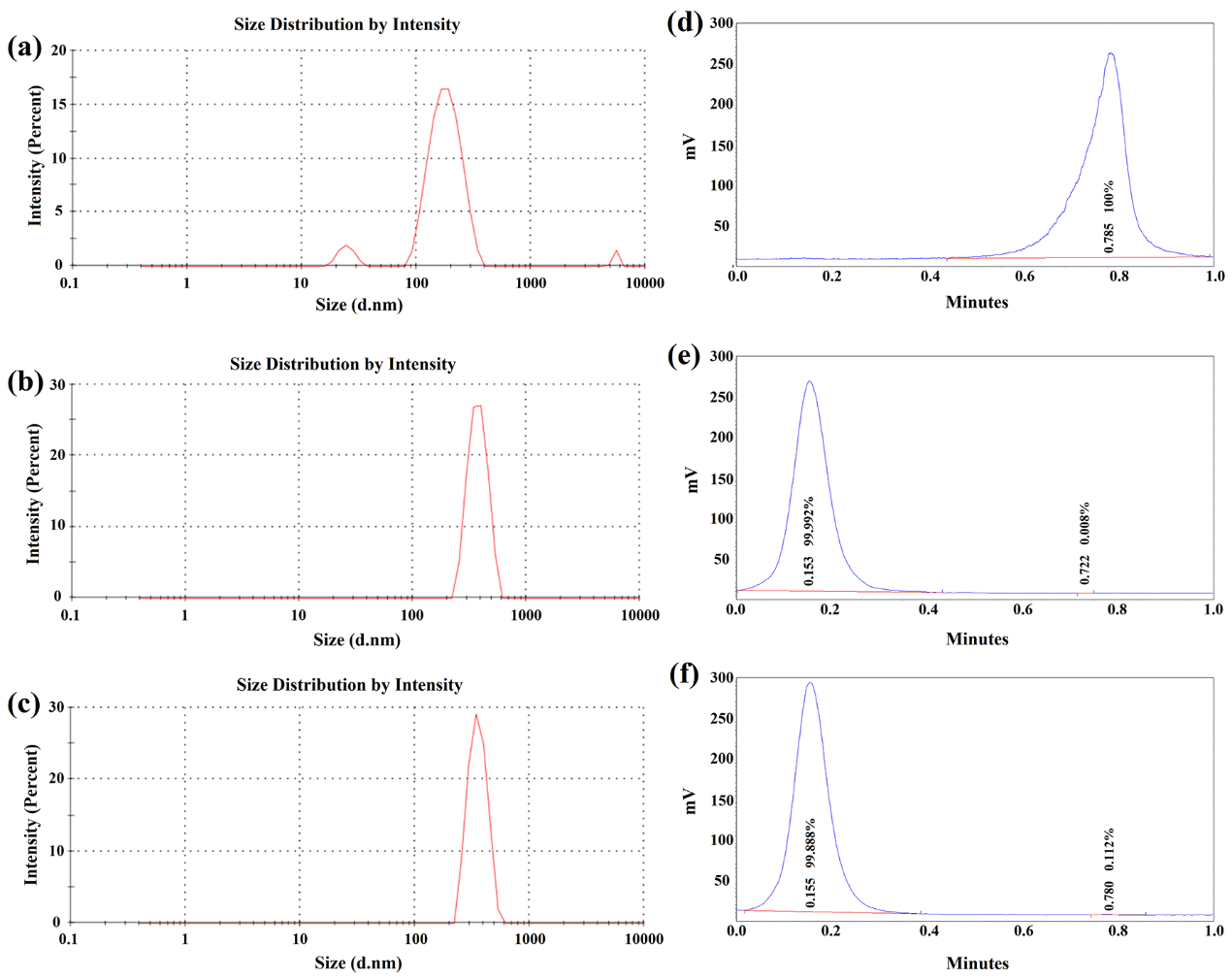


**Fig. S3** Hydrodynamic size distributions of **a** PEI.NH_2_-DTPA-(PEG-CTX)-*m*PEG, **b** *m*PEI/DOX, and **c** *m*PEI-CTX/DOX dispersed in water. ITLC results of **d** Na^99m^TcO_4_, **e** *m*PEI-^99m^Tc/DOX, and **f** *m*PEI-CTX-^99m^Tc/DOX on silica gel-coated fiber glass sheets using saline as the mobile phase.


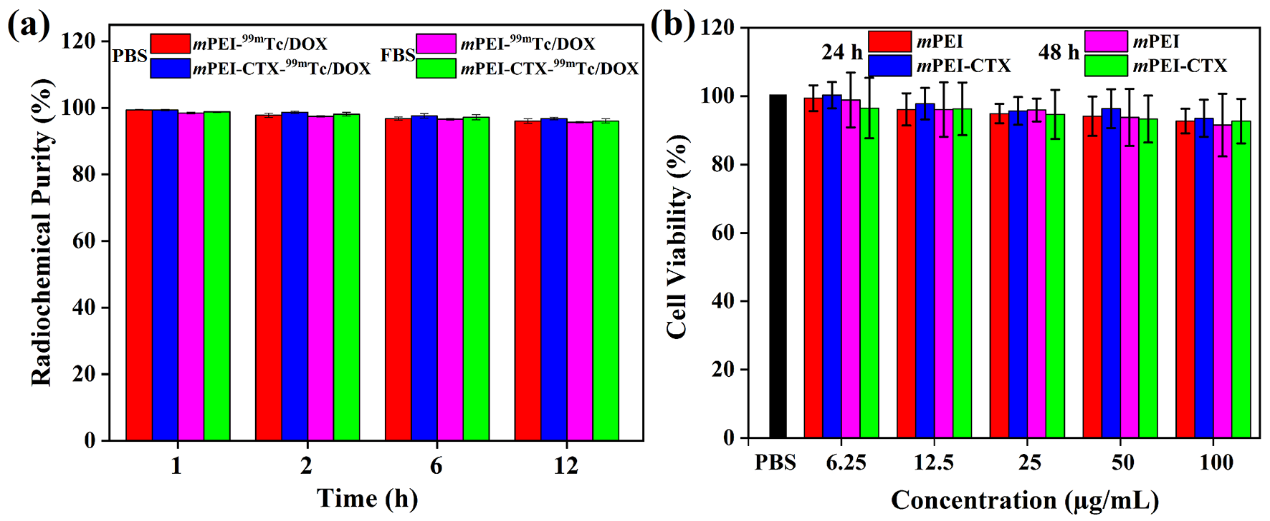


**Fig. S4** **a** Radiochemical purities of *m*PEI-CTX-^99m^Tc/DOX and *m*PEI-^99m^Tc/DOX in PBS at room temperature and FBS at 37 ºC for 1, 2, 6, and 12 h. **b** CCK-8 assay of C6 cells treated with the *m*PEI and *m*PEI-CTX at different polymer concentrations for 24 and 48 h, respectively.


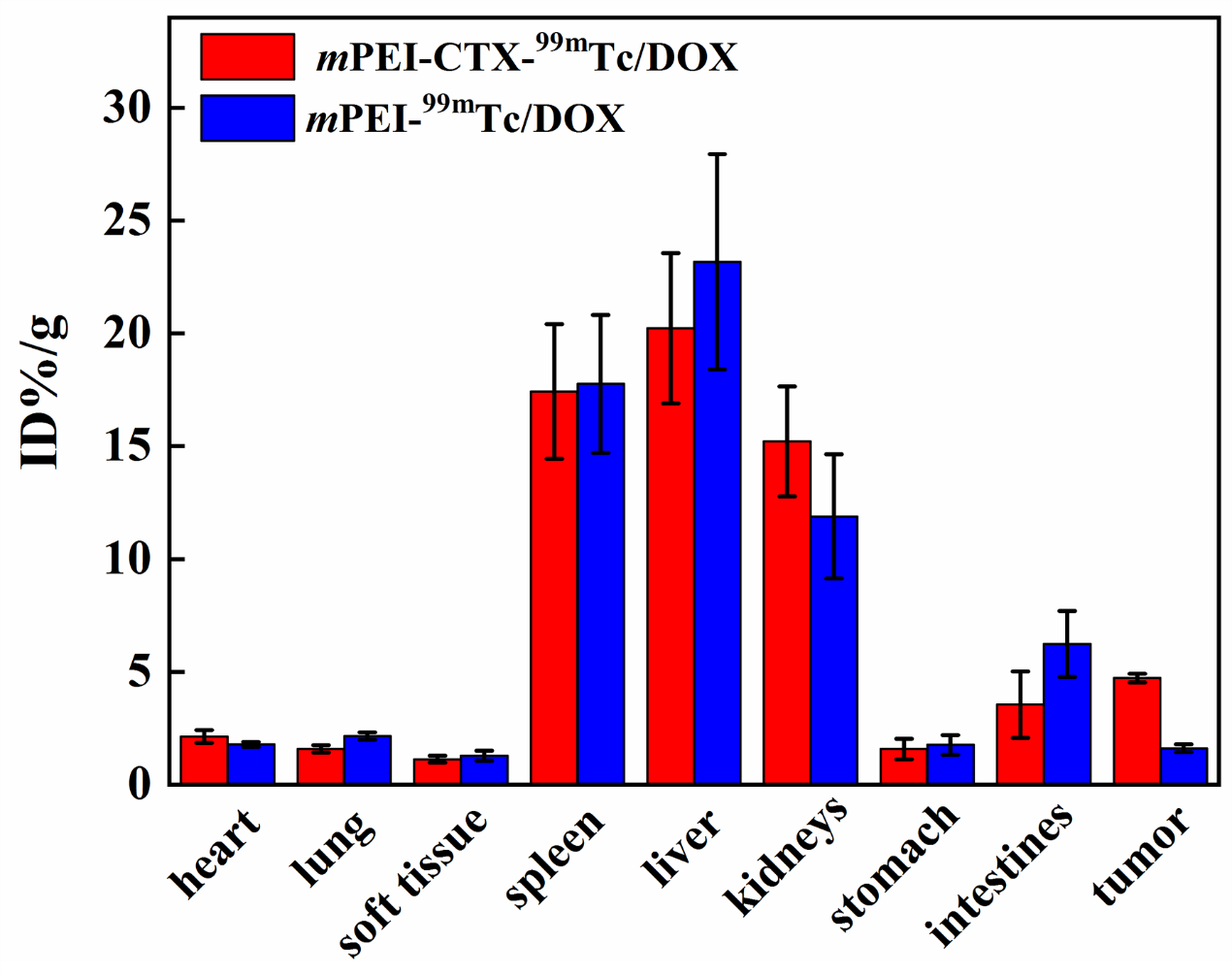


**Fig. S5** Biodistribution of the *m*PEI-CTX-^99m^Tc/DOX and *m*PEI-^99m^Tc/DOX at 12 h post-injection.





**Fig. S6** Body weight changes of the C6 tumor-bearing mice during treatments with *m*PEI-CTX/DOX, *m*PEI/DOX, *m*PEI-CTX, *m*PEI, DOX, and saline, respectively. The relative body weight was normalized according to their initial weights (Mean ± SD, n = 6).


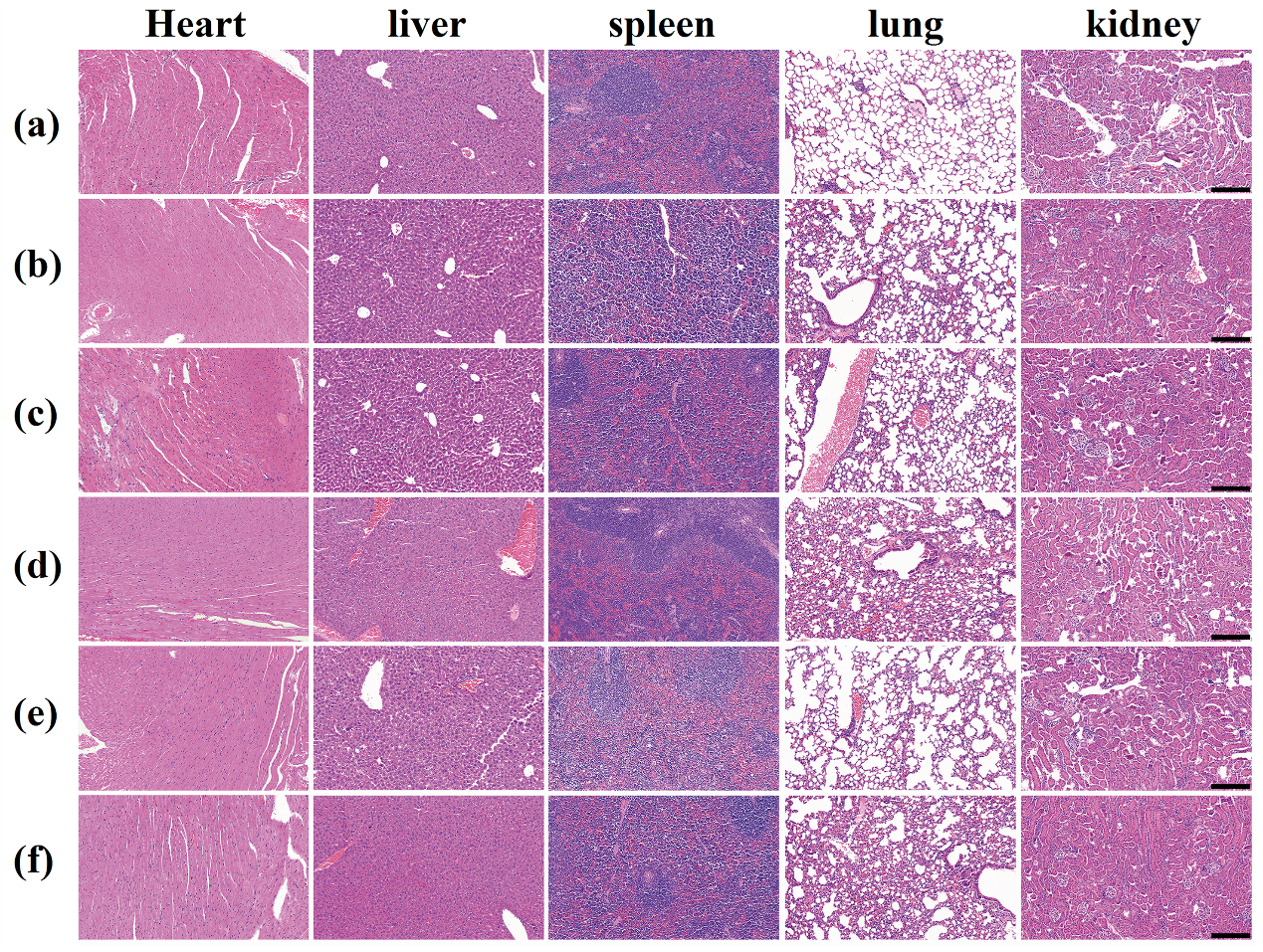


**Fig. S7** H&E staining of the heart, liver, spleen, lung, and kidney of the tumor-bearing mice after the 21-day treatment with **a** *m*PEI-CTX/DOX, **b** *m*PEI/DOX, **c** DOX, **d** *m*PEI-CTX, **e** *m*PEI, and **f** saline. The scale bar in each panel indicates 200 μm.
